# Supplementary material for: Non-criticality of interaction network over system’s crises: A percolation analysis
Source: Sci Rep. 2017 Nov 20;7:15855. doi: 10.1038/s41598-017-16223-6 (PMC5696469; doi:10.1038/s41598-017-16223-6)
Supplement: Supplementary file 1 — Supplementary Information [file 41598_2017_16223_MOESM1_ESM.pdf]

Non-criticality of interaction network over system's crises: A percolation analysis (Supplementary)

Amir Hossein Shirazi[1,*], **Abbas Ali Saberi**[2,3,4,+], **Ali Hosseiny**[1,4], **Ehsan Amirzadeh**[1], **and Pourya Toranj Simin**[1]

[1]Department of Physics, Shahid Beheshti University, G.C., Evin, Tehran 19839, Iran
[2]Department of Physics, University of Tehran, Tehran 14395-547, Iran
[3]Institut für Theoretische Physik, Universitat zu Köln, Zülpicher Strasse 77, 50937 Köln,Germany
[4]School of Physics and Accelerators, Institute for research in Fundamental Science (IPM) PO Box 19395-5531, Tehran, Iran
[*]amir.h.shirazi@gmail.com
[+]ab.saberi@ut.ac.ir

ABSTRACT

Giant component probability

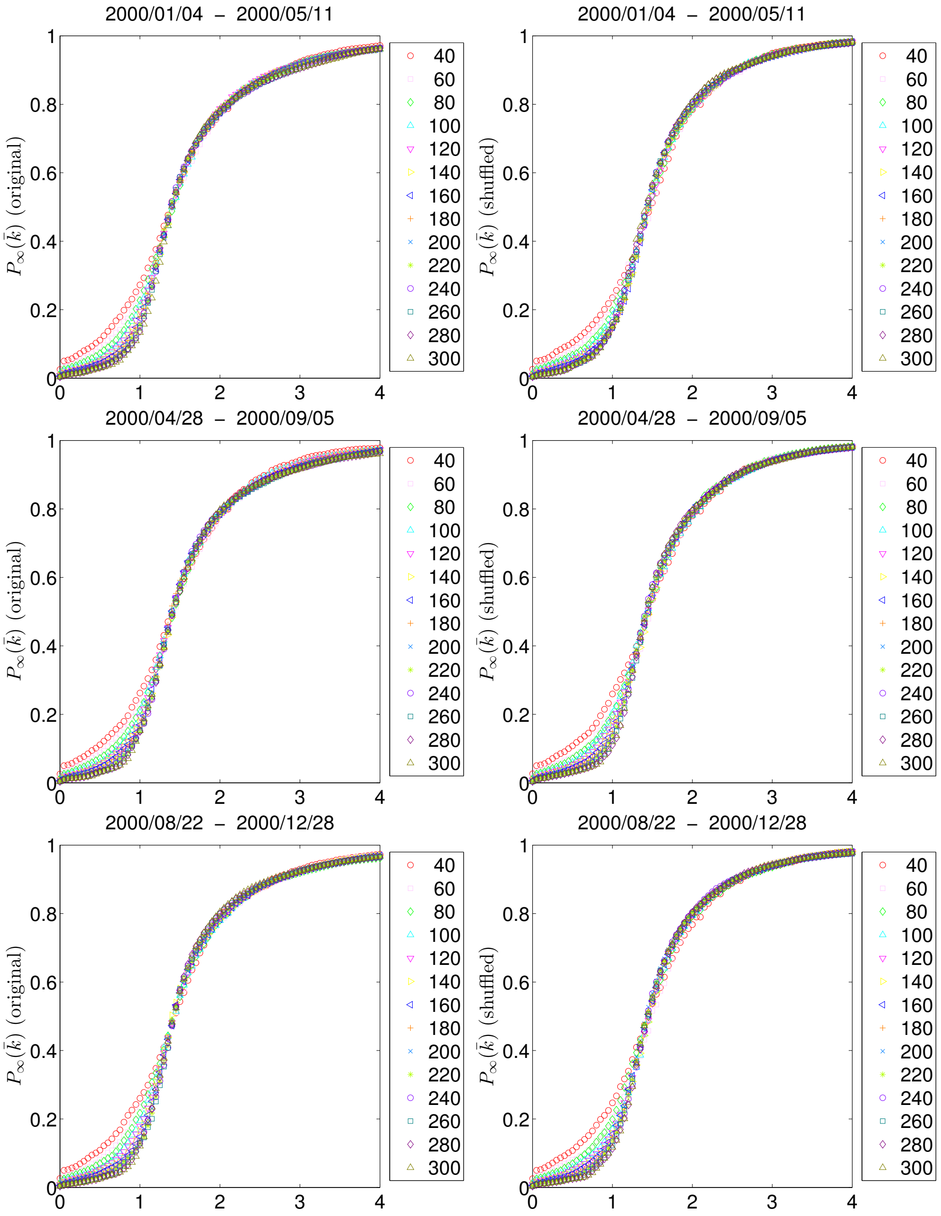
2000/01/04 – 2000/05/11
2000/04/28 – 2000/09/05
2000/08/22 – 2000/12/28
$P_\infty(\bar{k})$ (original)
$P_\infty(\bar{k})$ (shuffled)
40
60
80
100
120
140
160
180
200
220
240
260
280
300
0
0.2
0.4
0.6
0.8
1
2
3
4

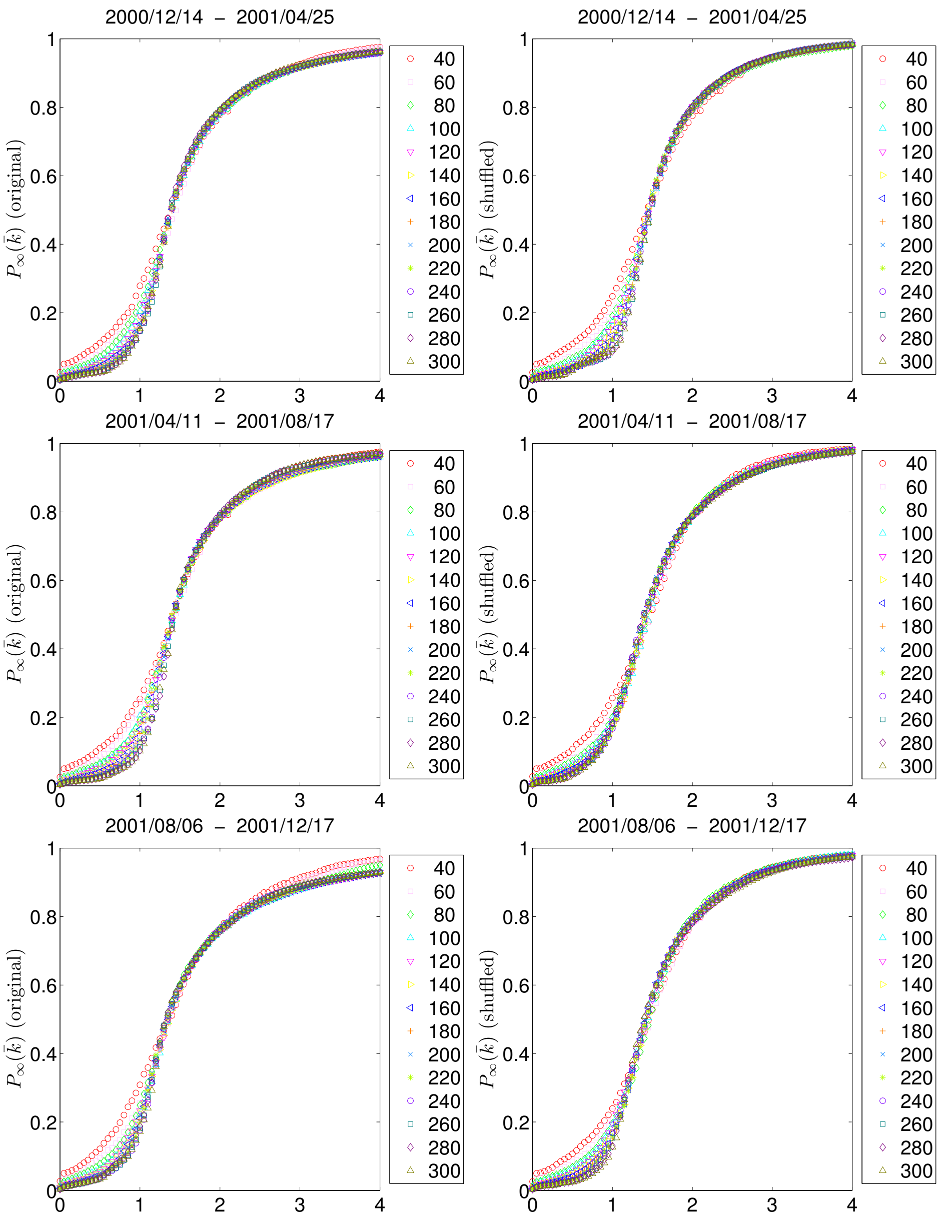
2000/12/14 – 2001/04/25
$P_\infty(\bar{k})$ (original)
$P_\infty(\bar{k})$ (shuffled)
2001/04/11 – 2001/08/17
$P_\infty(\bar{k})$ (original)
$P_\infty(\bar{k})$ (shuffled)
2001/08/06 – 2001/12/17
$P_\infty(\bar{k})$ (original)
$P_\infty(\bar{k})$ (shuffled)
40
60
80
100
120
140
160
180
200
220
240
260
280
300
0
0.2
0.4
0.6
0.8
1
0
1
2
3
4

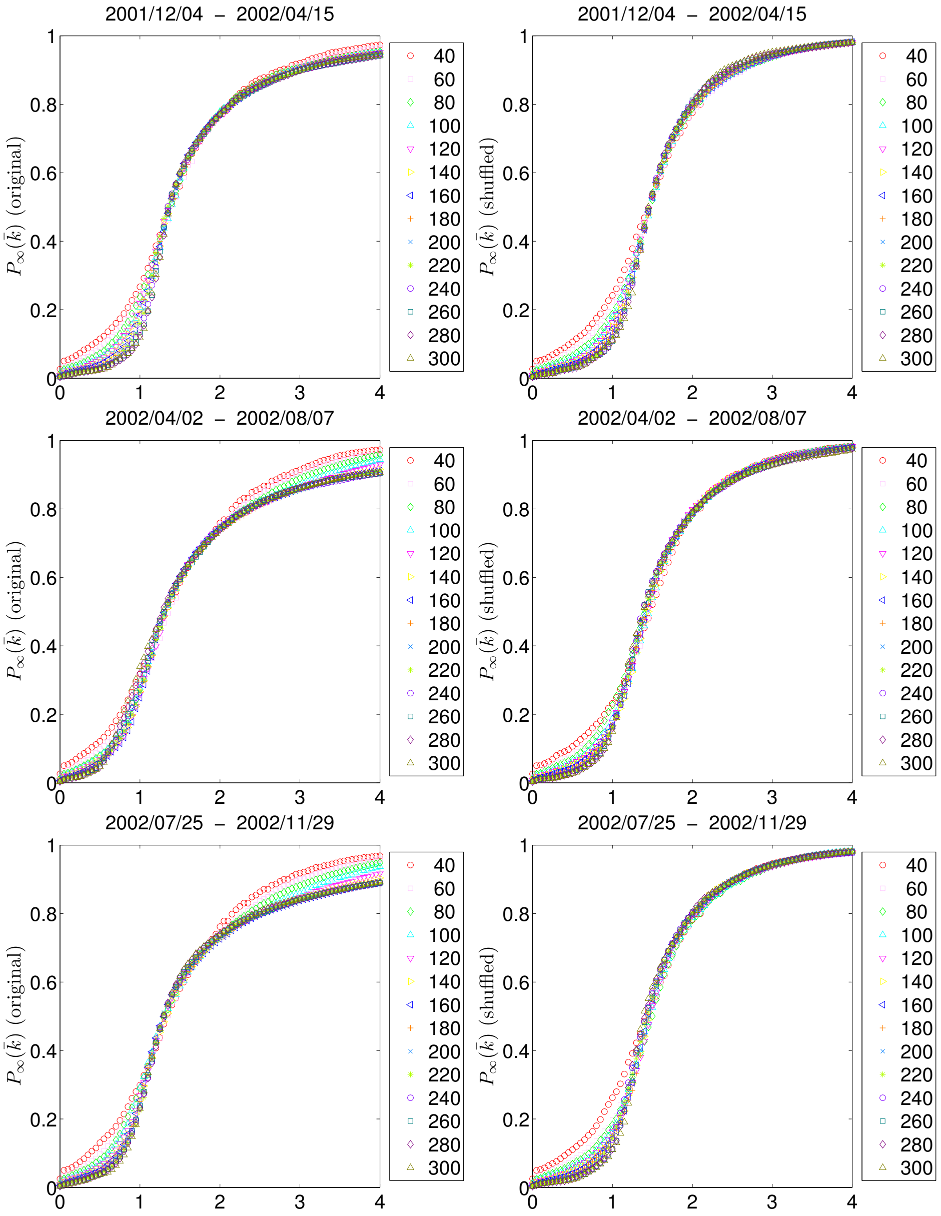

2001/12/04 – 2002/04/15
2001/12/04 – 2002/04/15
2002/04/02 – 2002/08/07
2002/04/02 – 2002/08/07
2002/07/25 – 2002/11/29
2002/07/25 – 2002/11/29
$P_\infty(\bar{k})$ (original)
$P_\infty(\bar{k})$ (shuffled)
40
60
80
100
120
140
160
180
200
220
240
260
280
300
0
0.2
0.4
0.6
0.8
1
0
1
2
3
4

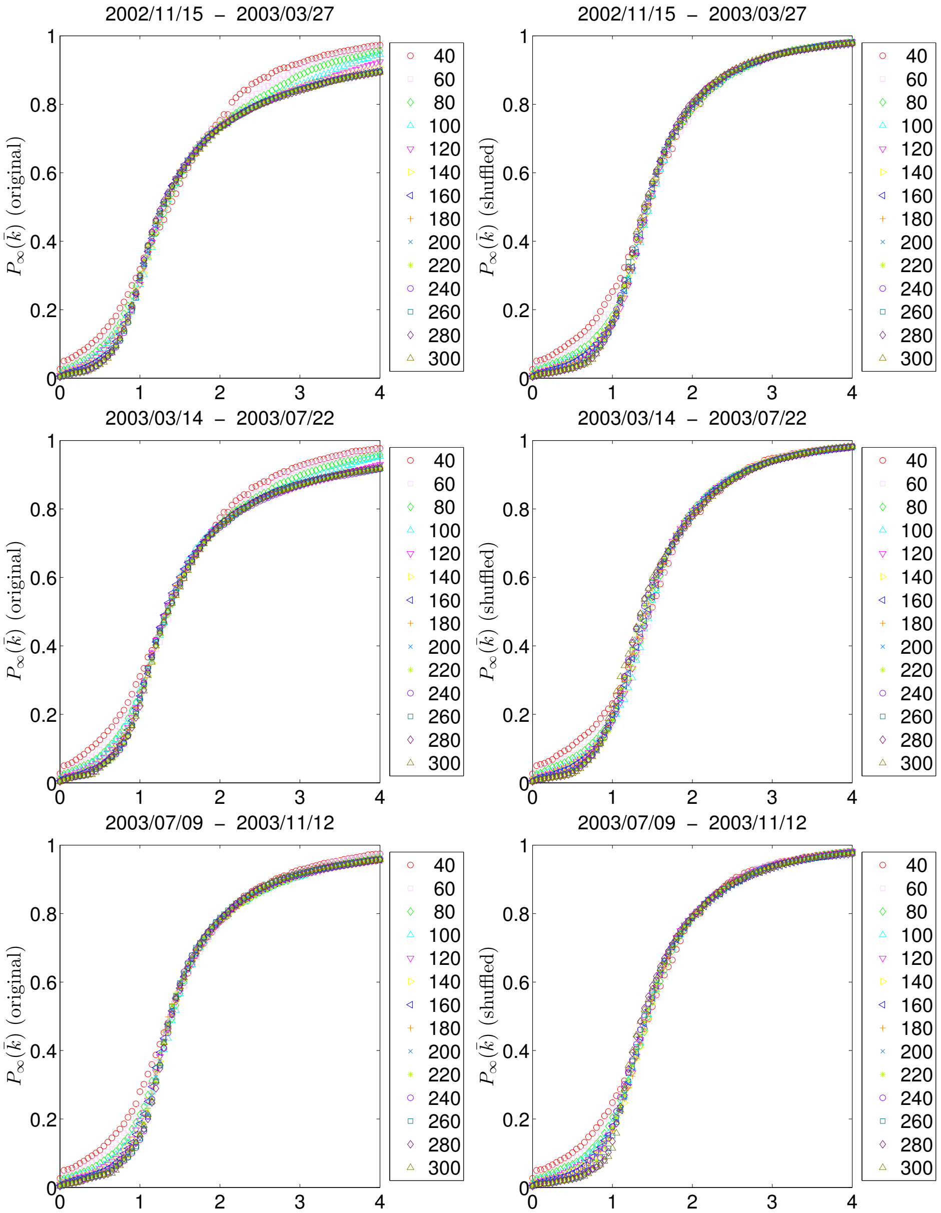

2002/11/15 – 2003/03/27
2003/03/14 – 2003/07/22
2003/07/09 – 2003/11/12
$P_\infty(\bar{k})$ (original)
$P_\infty(\bar{k})$ (shuffled)
40
60
80
100
120
140
160
180
200
220
240
260
280
300

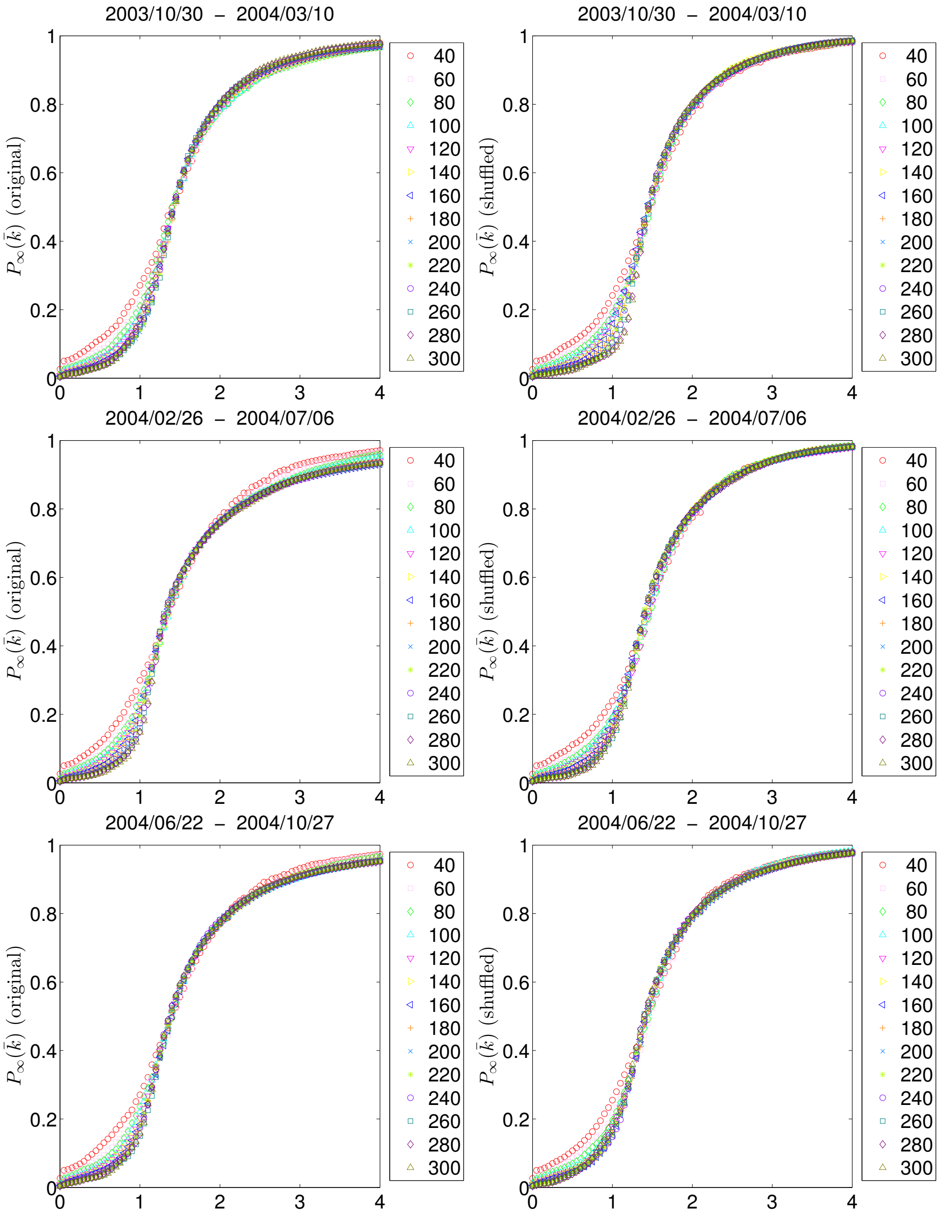
2003/10/30 – 2004/03/10
2004/02/26 – 2004/07/06
2004/06/22 – 2004/10/27
$P_\infty(\bar{k})$ (original)
$P_\infty(\bar{k})$ (shuffled)
40
60
80
100
120
140
160
180
200
220
240
260
280
300

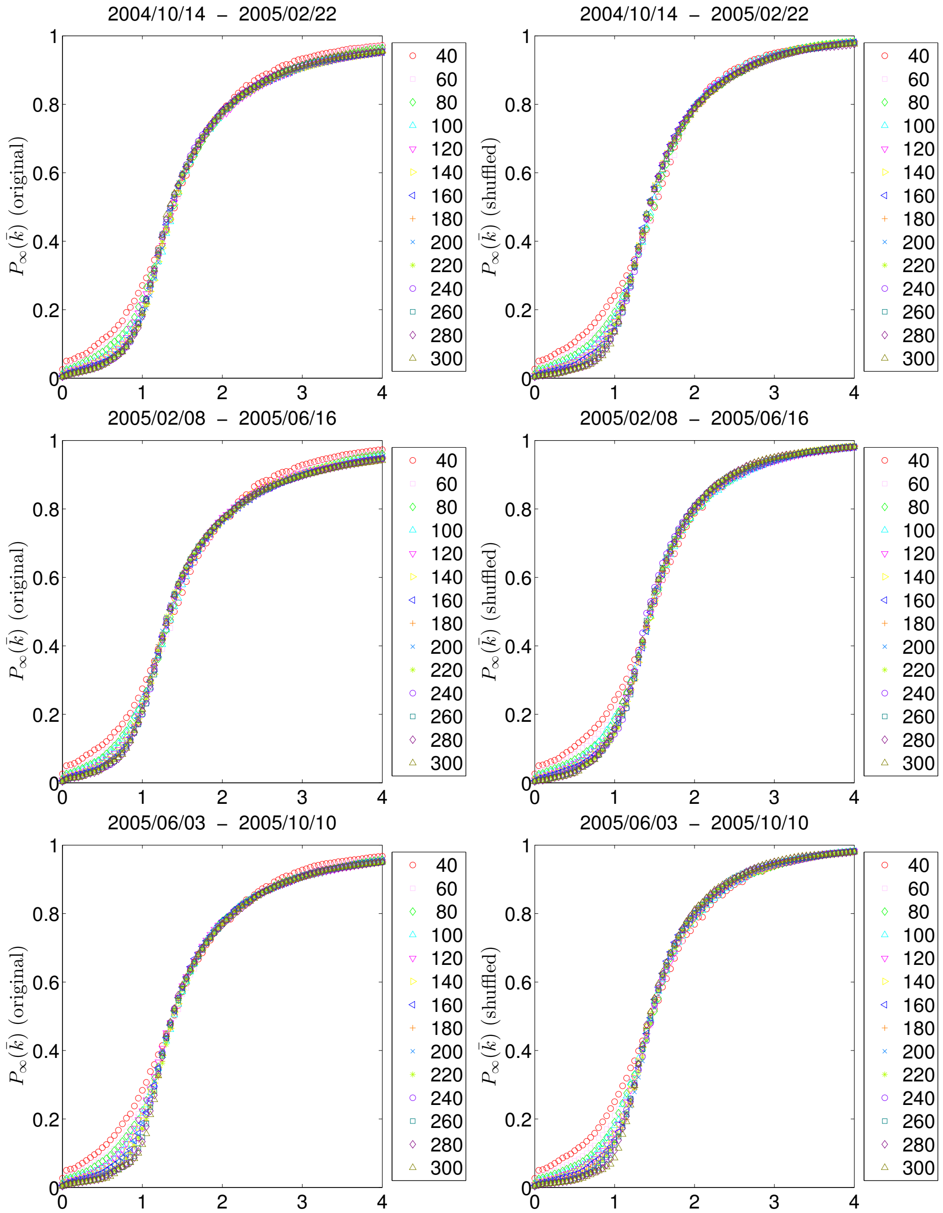
2004/10/14 – 2005/02/22
2004/10/14 – 2005/02/22
2005/02/08 – 2005/06/16
2005/02/08 – 2005/06/16
2005/06/03 – 2005/10/10
2005/06/03 – 2005/10/10
$P_\infty(\bar{k})$ (original)
$P_\infty(\bar{k})$ (shuffled)
0
0.2
0.4
0.6
0.8
1
0
1
2
3
4
40
60
80
100
120
140
160
180
200
220
240
260
280
300

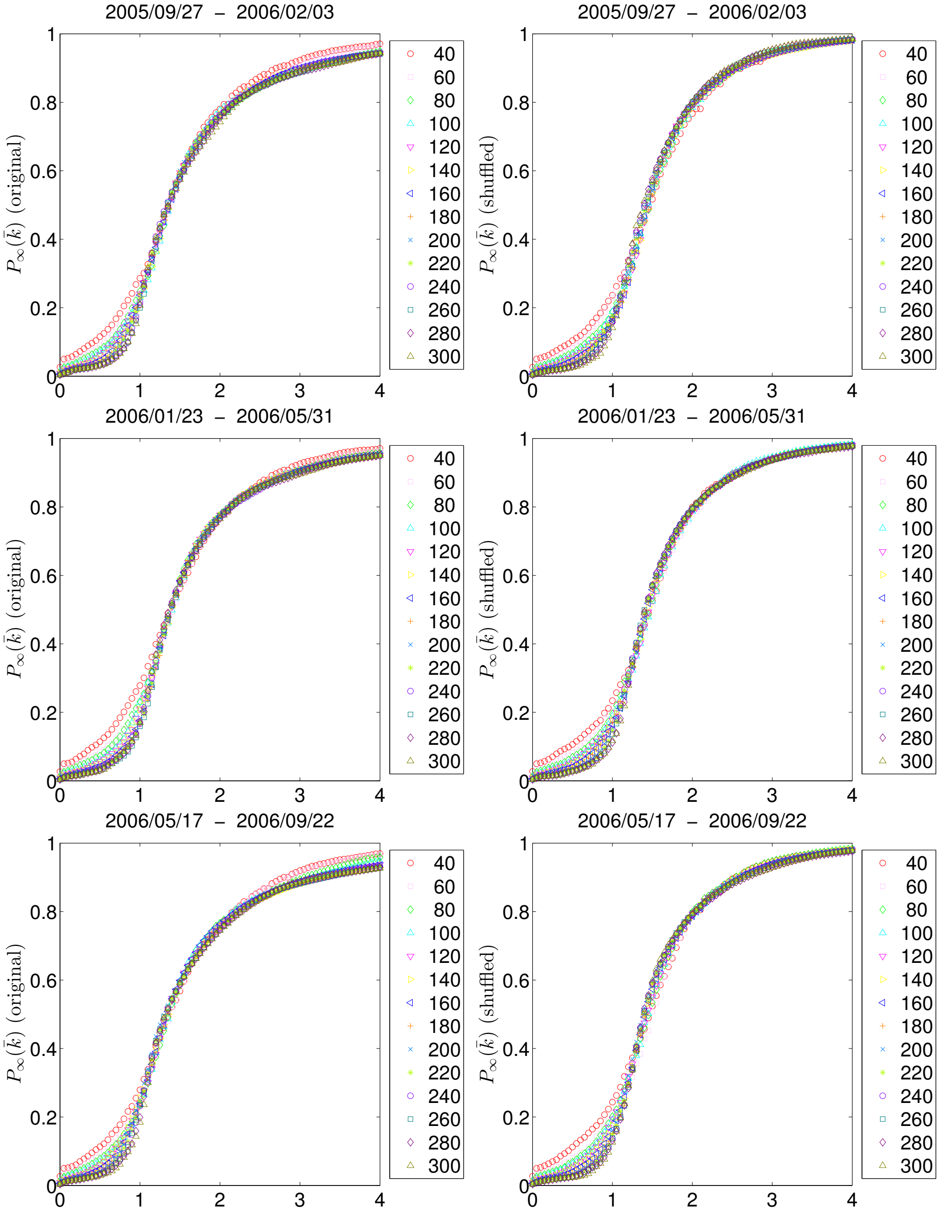

2005/09/27 – 2006/02/03
2006/01/23 – 2006/05/31
2006/05/17 – 2006/09/22
$P_\infty(\bar{k})$ (original)
$P_\infty(\bar{k})$ (shuffled)
40
60
80
100
120
140
160
180
200
220
240
260
280
300

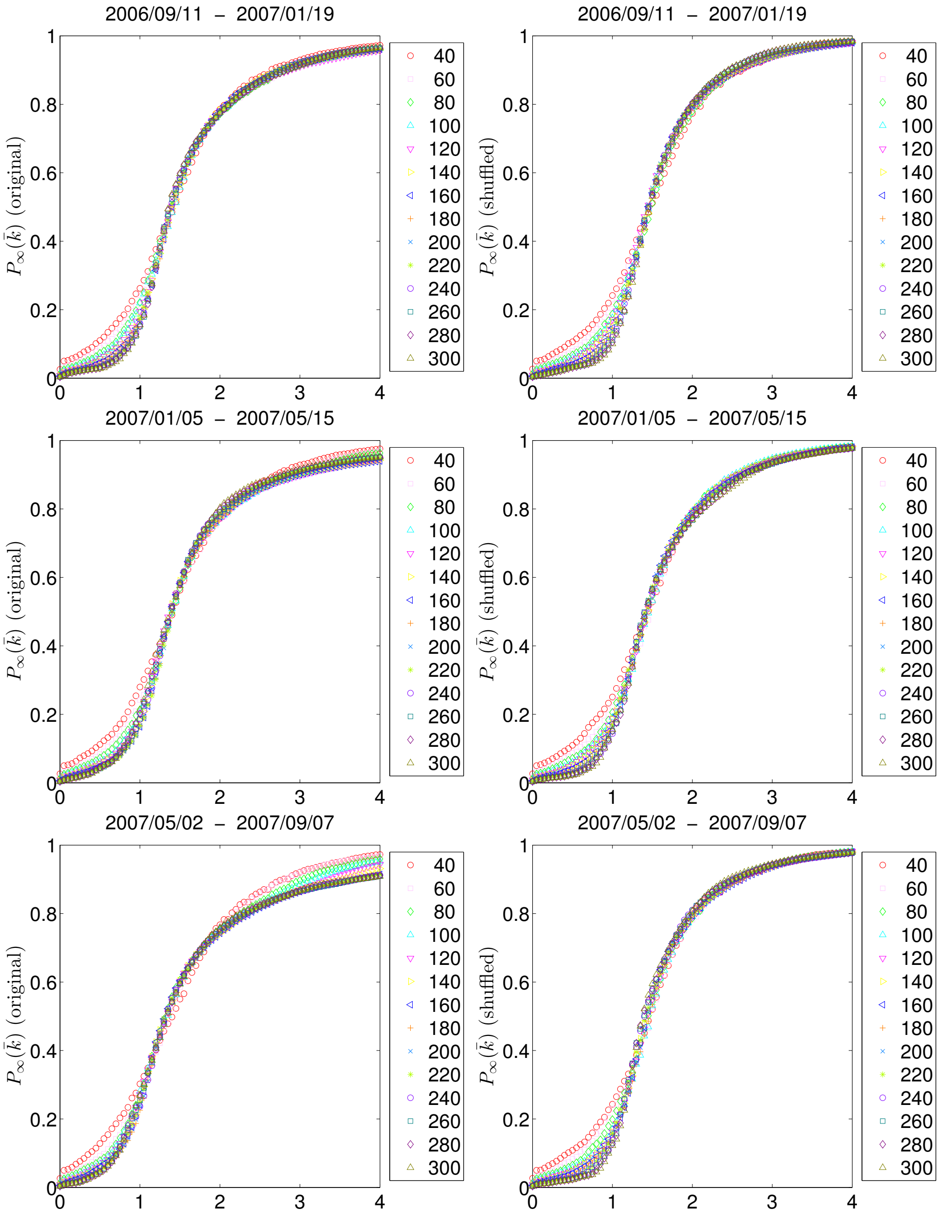
2006/09/11 – 2007/01/19
2007/01/05 – 2007/05/15
2007/05/02 – 2007/09/07
$P_\infty(\bar{k})$ (original)
$P_\infty(\bar{k})$ (shuffled)
40
60
80
100
120
140
160
180
200
220
240
260
280
300

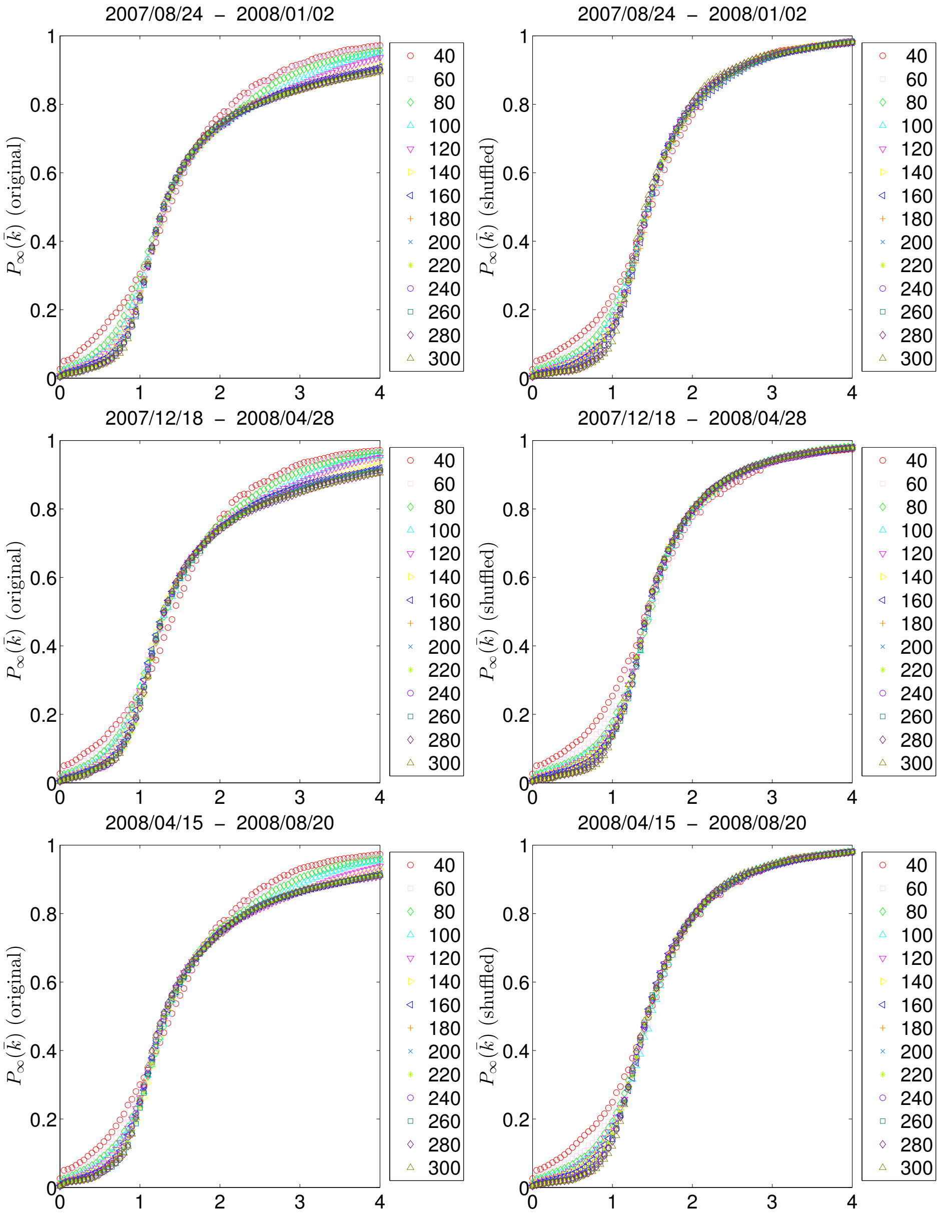
2007/08/24 – 2008/01/02
$P_\infty(\bar{k})$ (original)
$P_\infty(\bar{k})$ (shuffled)
2007/12/18 – 2008/04/28
$P_\infty(\bar{k})$ (original)
$P_\infty(\bar{k})$ (shuffled)
2008/04/15 – 2008/08/20
$P_\infty(\bar{k})$ (original)
$P_\infty(\bar{k})$ (shuffled)
0 0.2 0.4 0.6 0.8 1
0 1 2 3 4
40 60 80 100 120 140 160 180 200 220 240 260 280 300

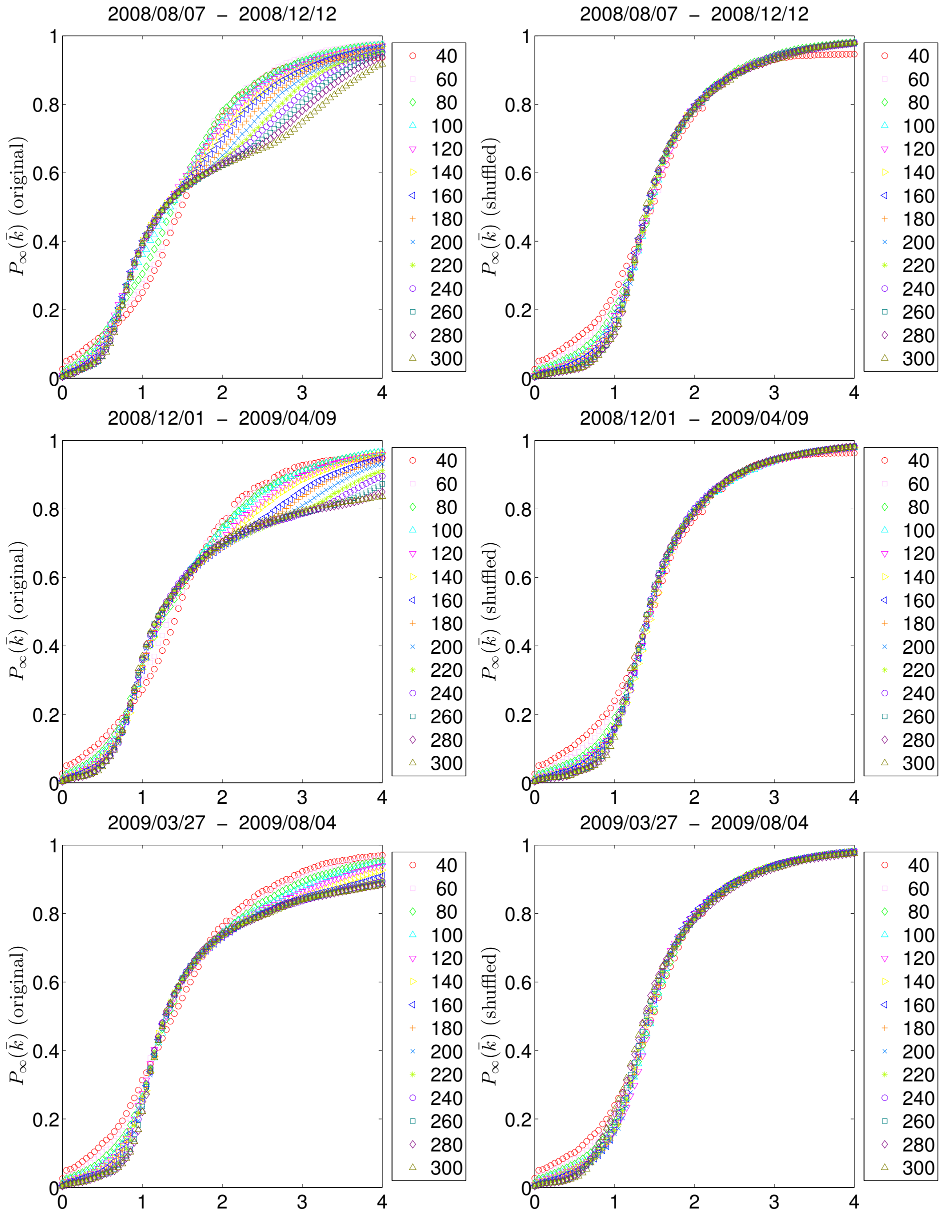
2008/08/07 – 2008/12/12
2008/12/01 – 2009/04/09
2009/03/27 – 2009/08/04
$P_\infty(\bar{k})$ (original)
$P_\infty(\bar{k})$ (shuffled)
40
60
80
100
120
140
160
180
200
220
240
260
280
300
0
0.2
0.4
0.6
0.8
1
0
1
2
3
4

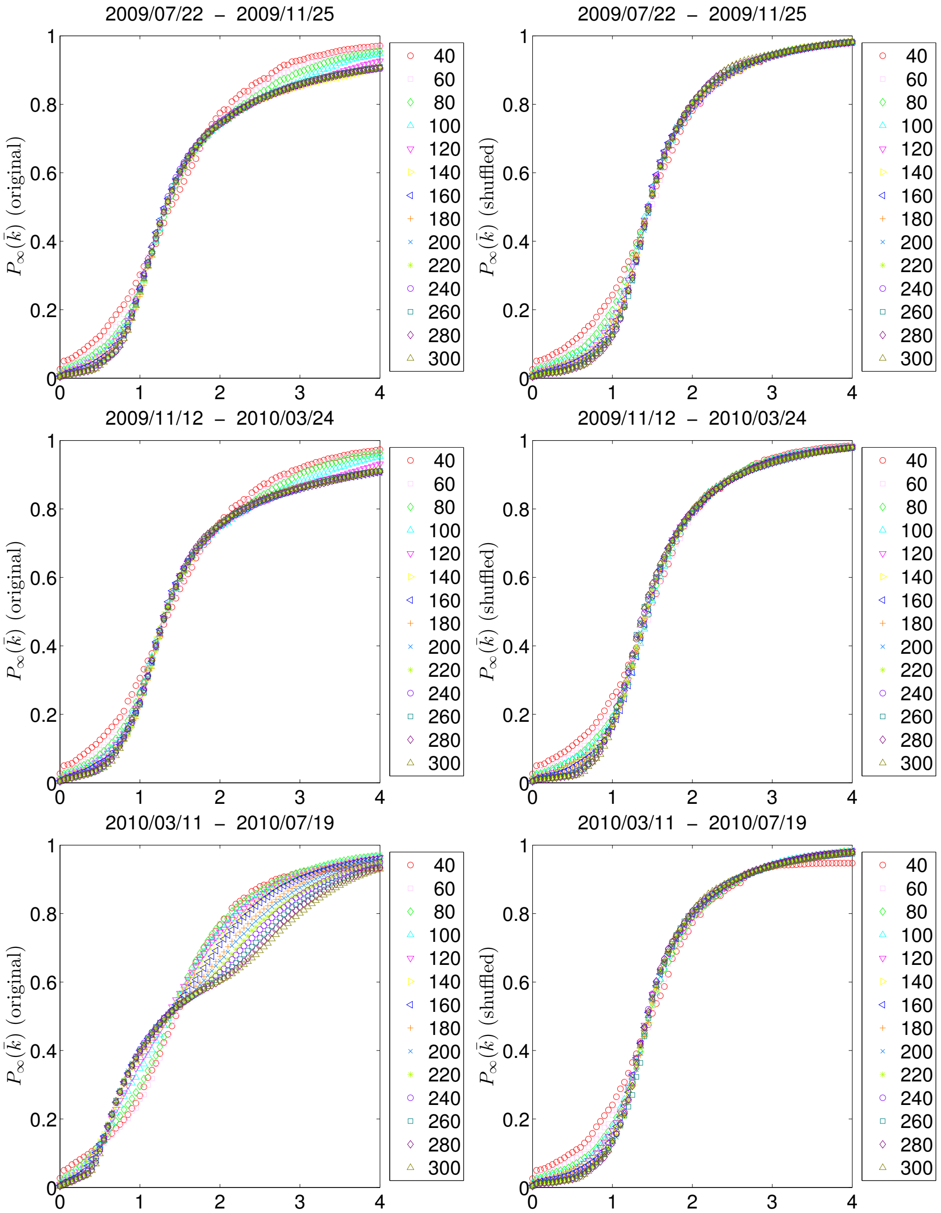
2009/07/22 – 2009/11/25
2009/11/12 – 2010/03/24
2010/03/11 – 2010/07/19
$P_\infty(\bar{k})$ (original)
$P_\infty(\bar{k})$ (shuffled)
40
60
80
100
120
140
160
180
200
220
240
260
280
300

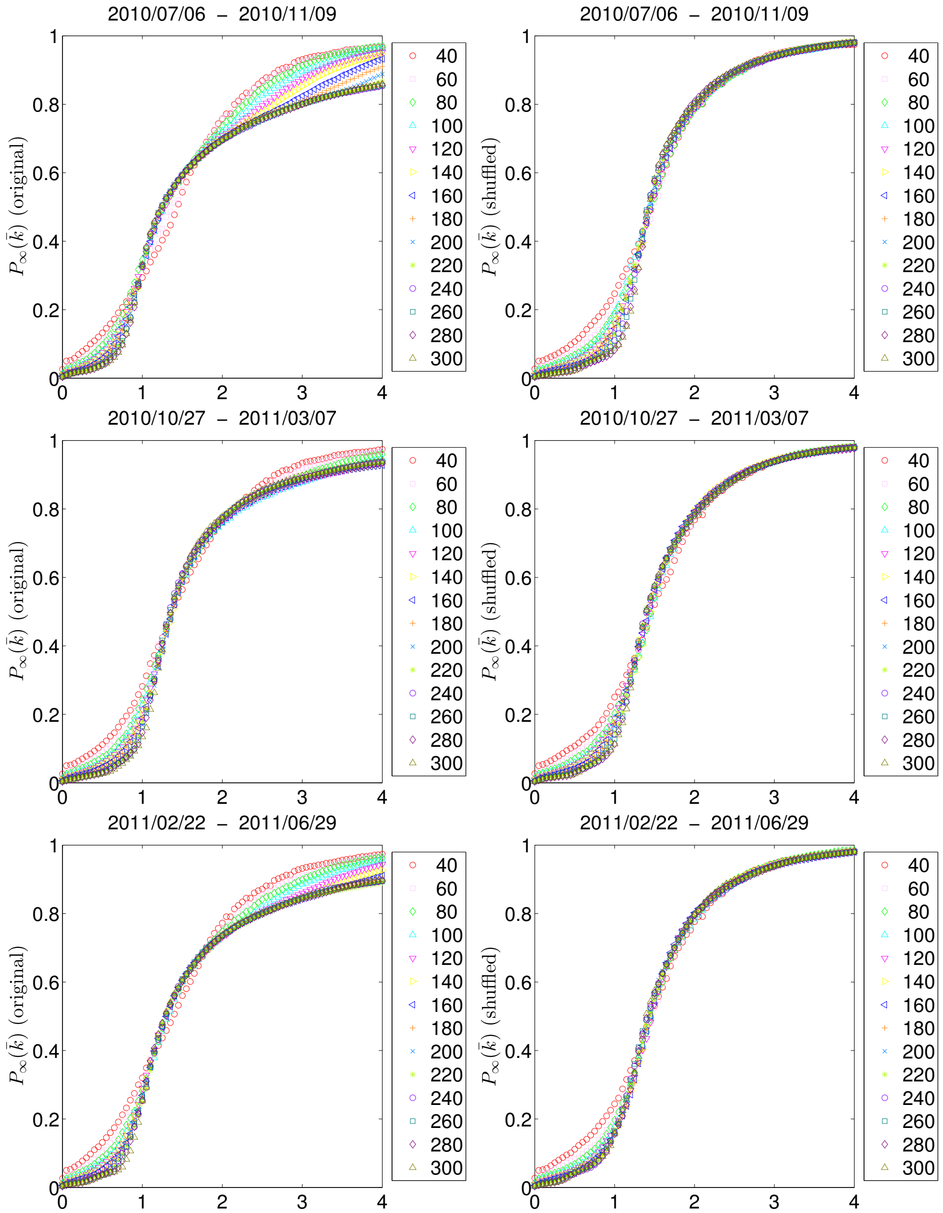

2010/07/06 – 2010/11/09
2010/10/27 – 2011/03/07
2011/02/22 – 2011/06/29
$P_\infty(\bar{k})$ (original)
$P_\infty(\bar{k})$ (shuffled)
40
60
80
100
120
140
160
180
200
220
240
260
280
300

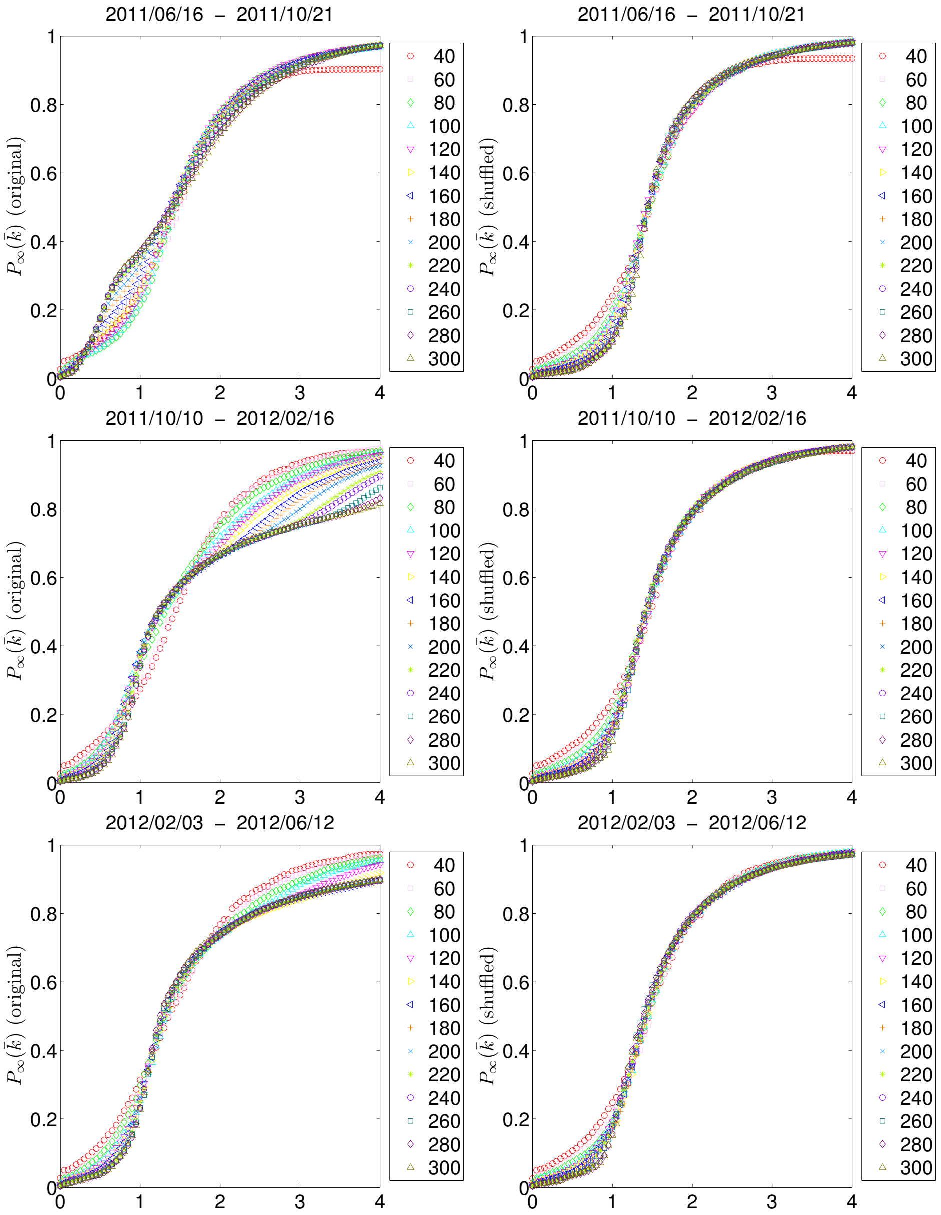

2011/06/16 – 2011/10/21
2011/10/10 – 2012/02/16
2012/02/03 – 2012/06/12
$P_\infty(\bar{k})$ (original)
$P_\infty(\bar{k})$ (shuffled)
40
60
80
100
120
140
160
180
200
220
240
260
280
300

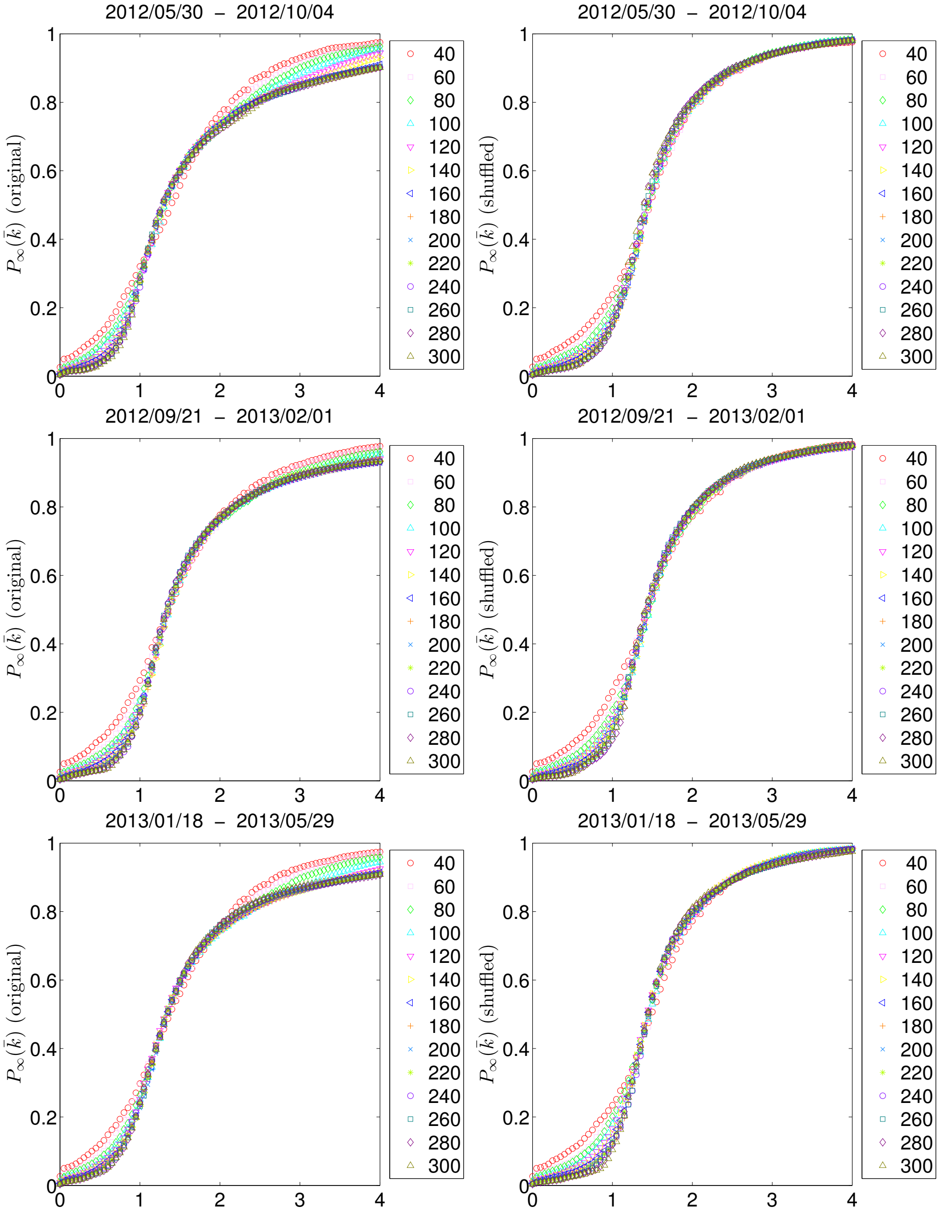
2012/05/30 – 2012/10/04
2012/09/21 – 2013/02/01
2013/01/18 – 2013/05/29
$P_\infty(\bar{k})$ (original)
$P_\infty(\bar{k})$ (shuffled)
40
60
80
100
120
140
160
180
200
220
240
260
280
300

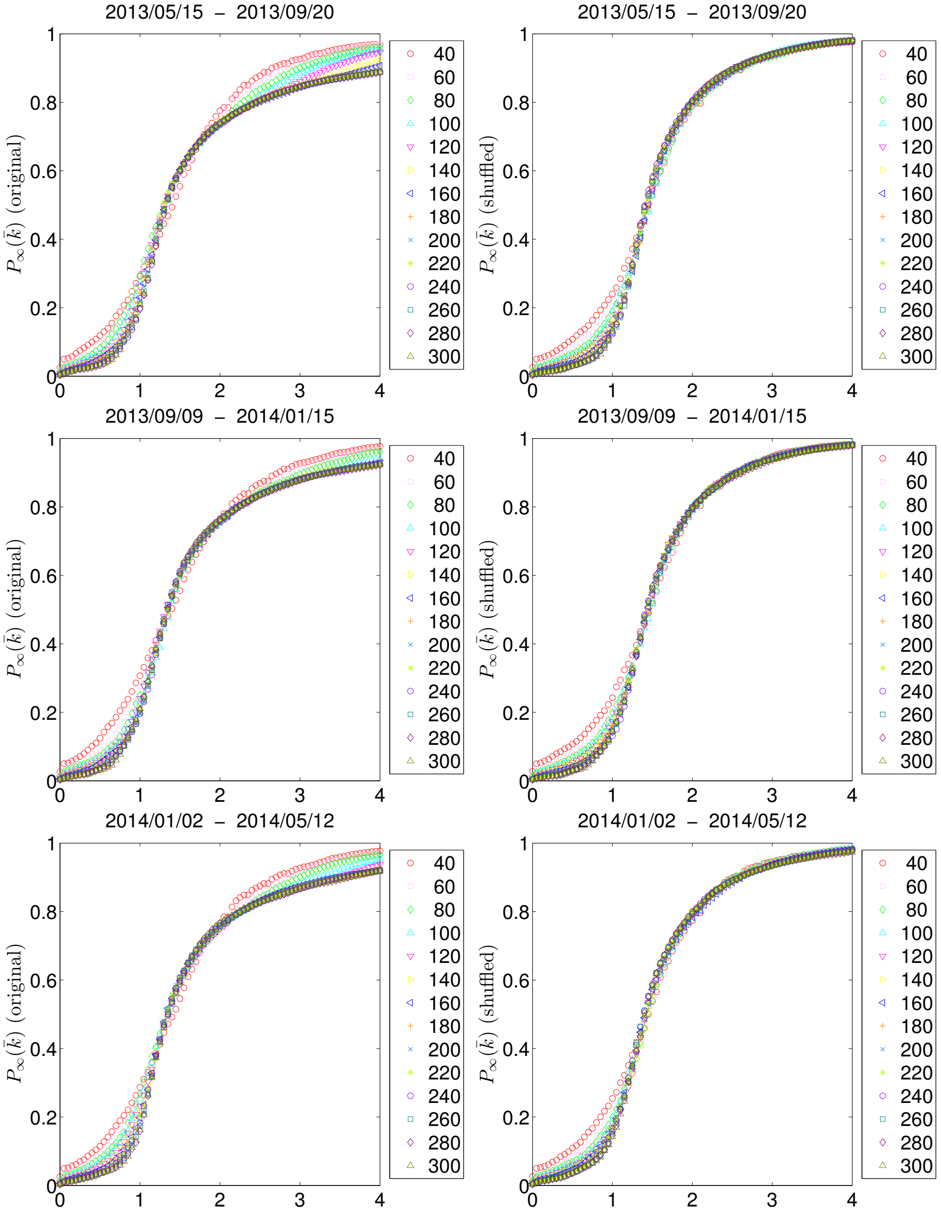
2013/05/15 – 2013/09/20
2013/09/09 – 2014/01/15
2014/01/02 – 2014/05/12
$P_\infty(\bar{k})$ (original)
$P_\infty(\bar{k})$ (shuffled)
40
60
80
100
120
140
160
180
200
220
240
260
280
300

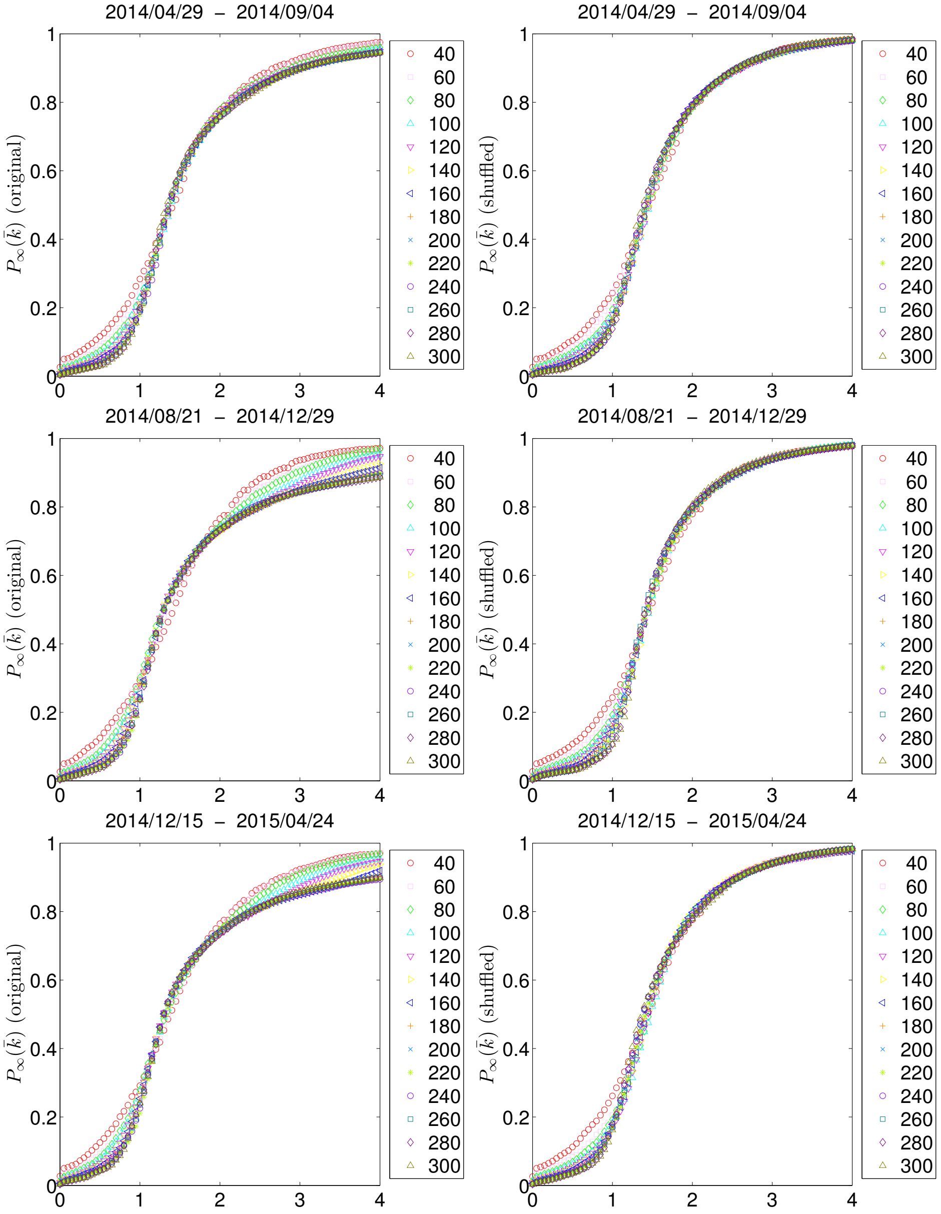
2014/04/29 – 2014/09/04
2014/08/21 – 2014/12/29
2014/12/15 – 2015/04/24
$P_\infty(\bar{k})$ (original)
$P_\infty(\bar{k})$ (shuffled)
40
60
80
100
120
140
160
180
200
220
240
260
280
300

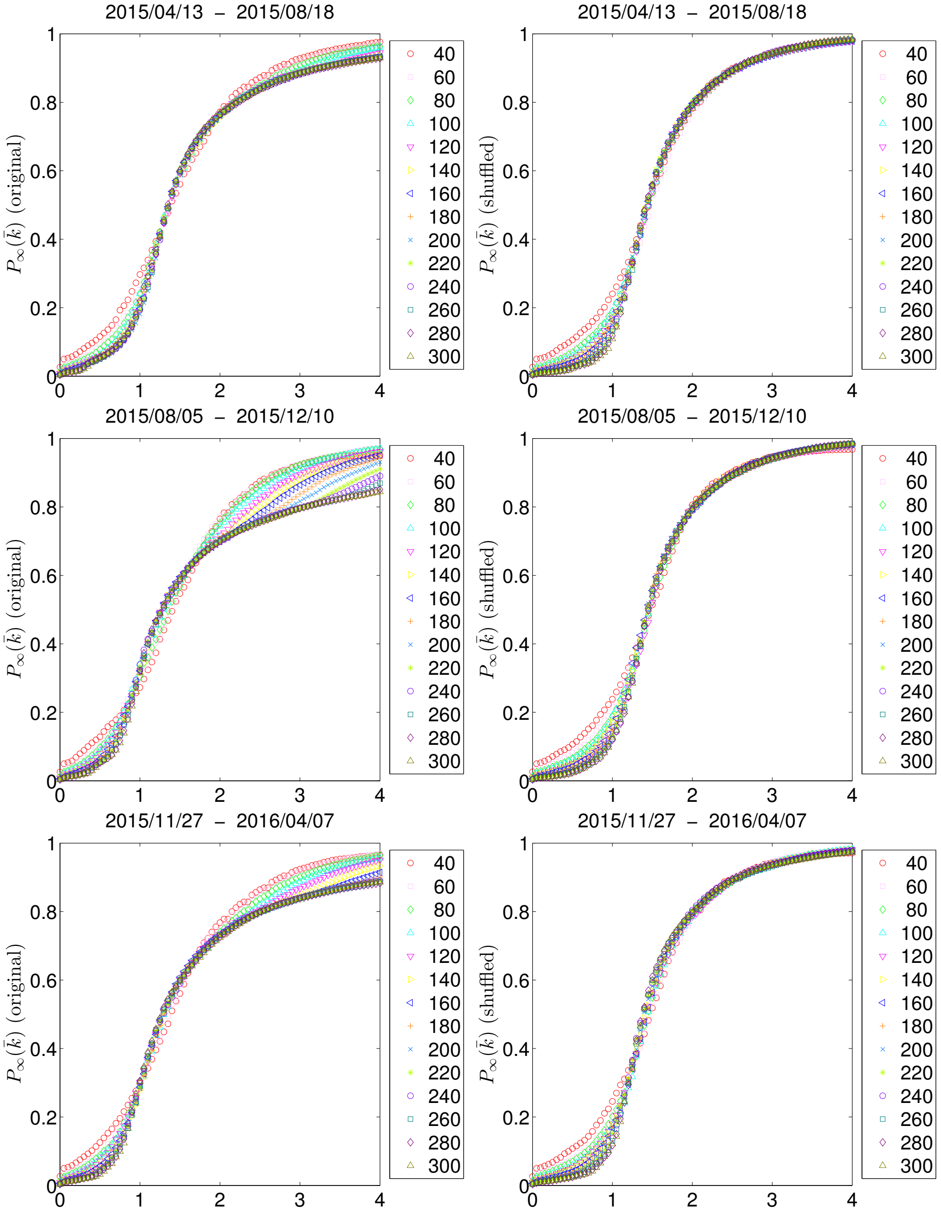
2015/04/13 – 2015/08/18
$P_\infty(\bar{k})$ (original)
$P_\infty(\bar{k})$ (shuffled)
2015/08/05 – 2015/12/10
2015/11/27 – 2016/04/07
40
60
80
100
120
140
160
180
200
220
240
260
280
300
0
0.2
0.4
0.6
0.8
1
2
3
4

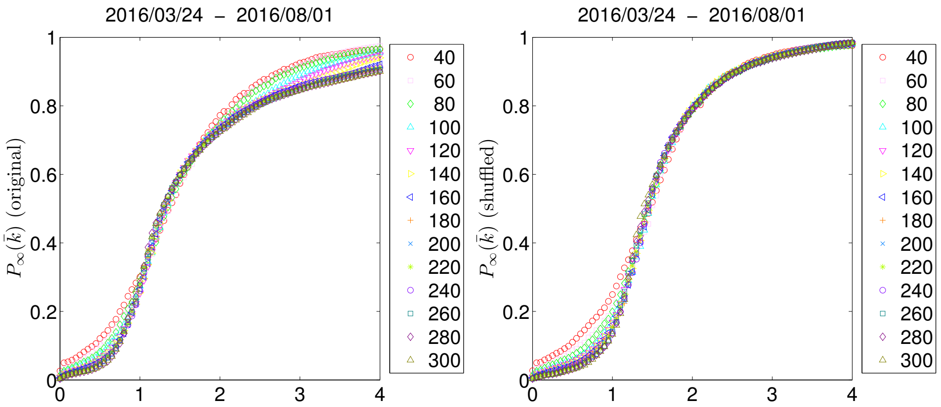

2016/03/24 – 2016/08/01
$P_\infty(\bar{k})$ (original)
$P_\infty(\bar{k})$ (shuffled)
40
60
80
100
120
140
160
180
200
220
240
260
280
300

Mean cluster size

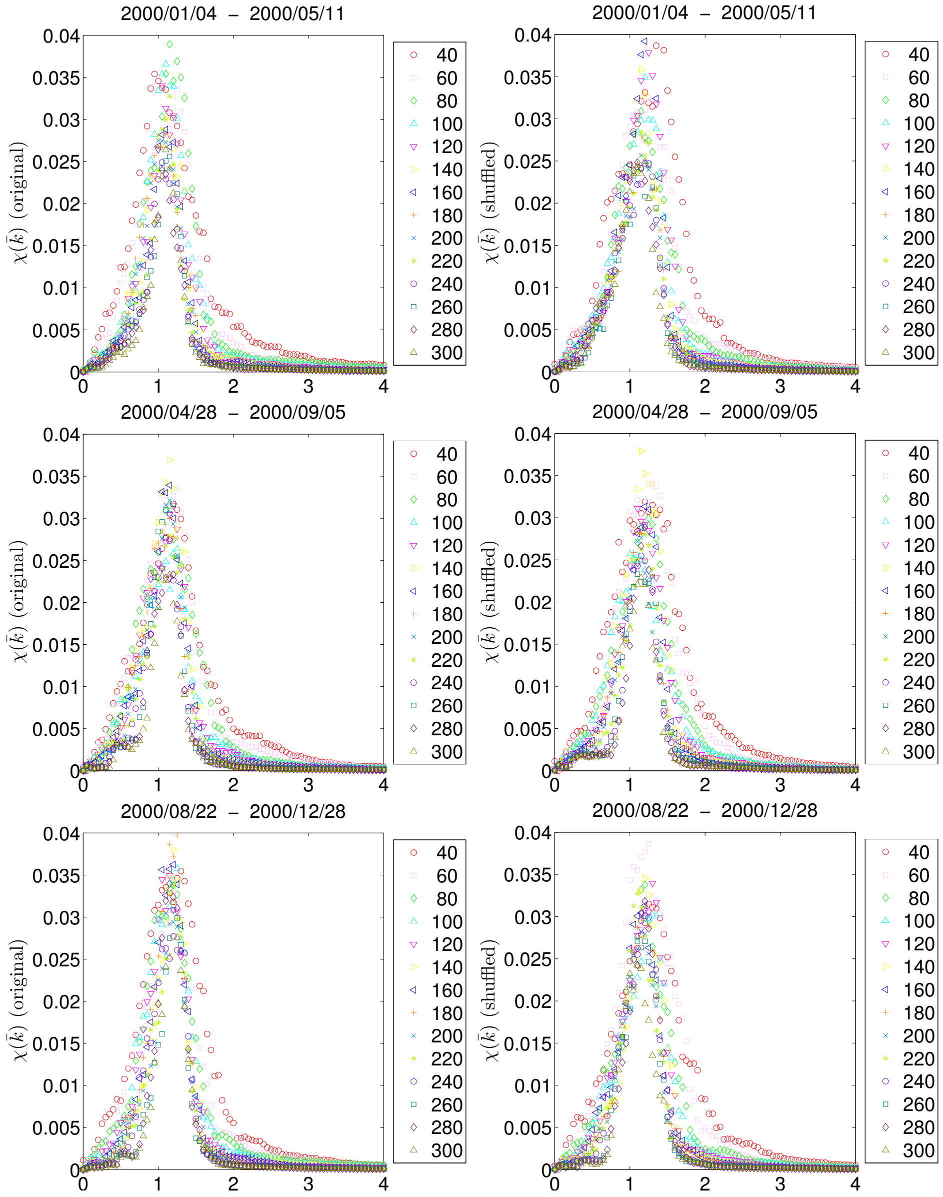
2000/01/04 – 2000/05/11
2000/04/28 – 2000/09/05
2000/08/22 – 2000/12/28
$\chi(\bar{k})$ (original)
$\chi(\bar{k})$ (shuffled)
40
60
80
100
120
140
160
180
200
220
240
260
280
300

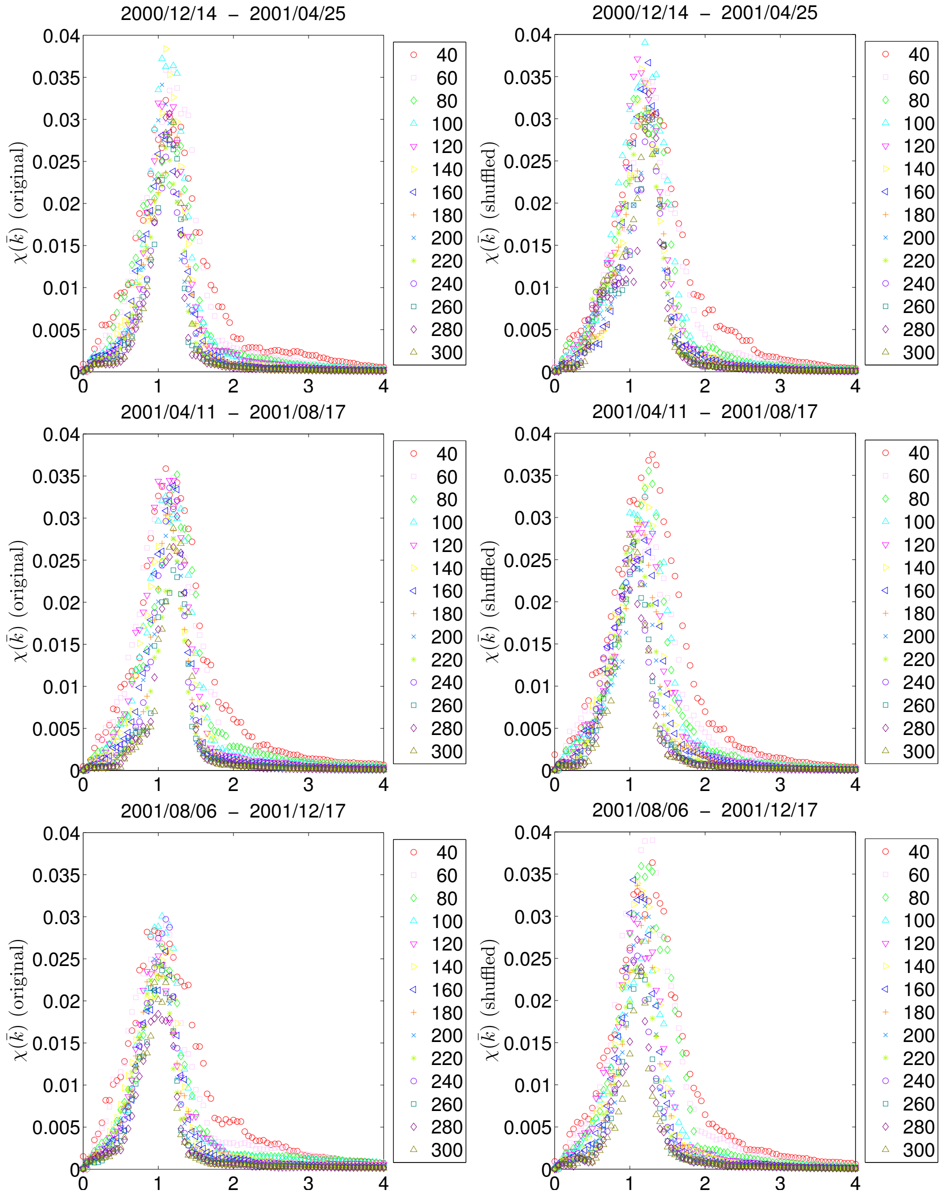
2000/12/14 – 2001/04/25
$\chi(\bar{k})$ (original)
2000/12/14 – 2001/04/25
$\chi(\bar{k})$ (shuffled)
2001/04/11 – 2001/08/17
$\chi(\bar{k})$ (original)
2001/04/11 – 2001/08/17
$\chi(\bar{k})$ (shuffled)
2001/08/06 – 2001/12/17
$\chi(\bar{k})$ (original)
2001/08/06 – 2001/12/17
$\chi(\bar{k})$ (shuffled)
0.04
0.035
0.03
0.025
0.02
0.015
0.01
0.005
0
0 1 2 3 4
40 60 80 100 120 140 160 180 200 220 240 260 280 300

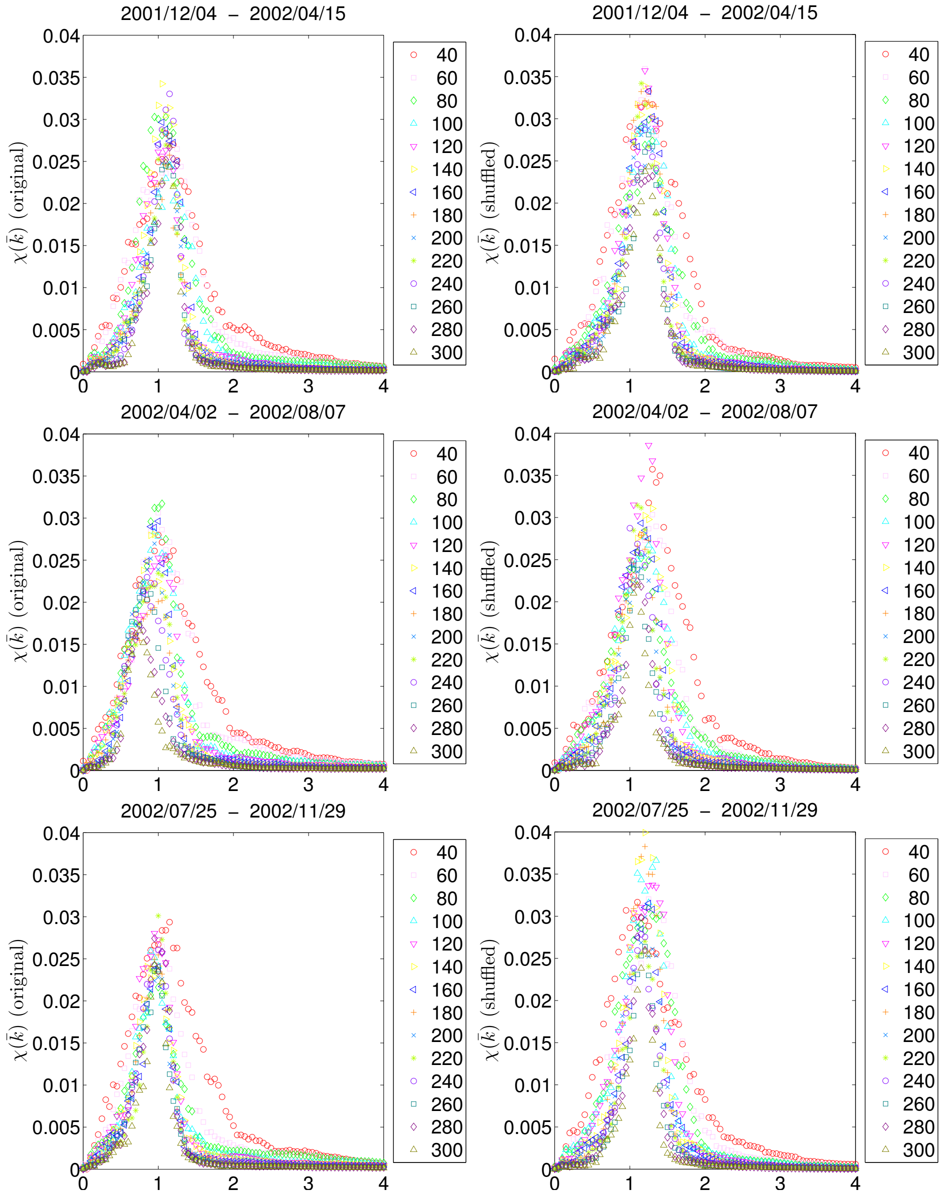
2001/12/04 – 2002/04/15
2002/04/02 – 2002/08/07
2002/07/25 – 2002/11/29
$\chi(\bar{k})$ (original)
$\chi(\bar{k})$ (shuffled)
40
60
80
100
120
140
160
180
200
220
240
260
280
300

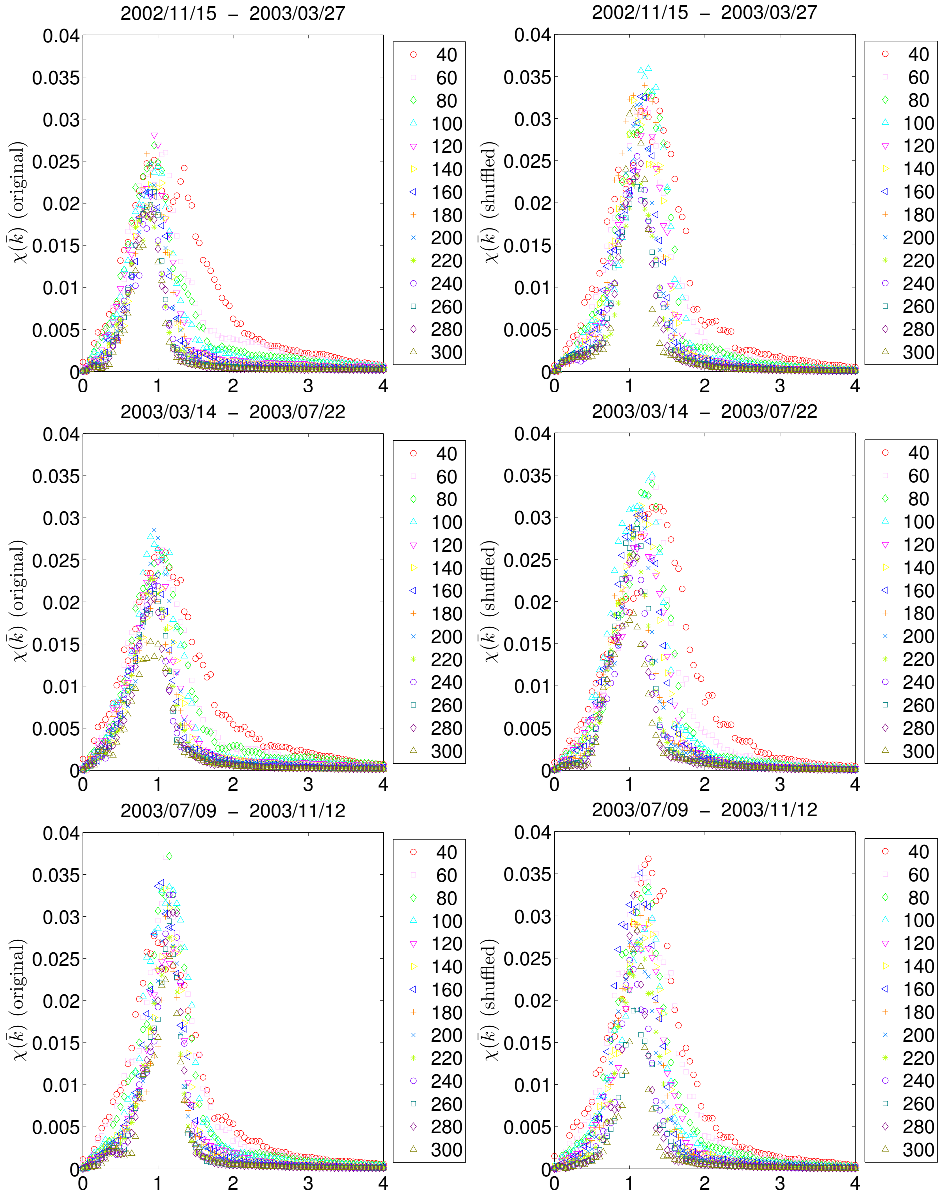
2002/11/15 – 2003/03/27
$\chi(\bar{k})$ (original)
$\chi(\bar{k})$ (shuffled)
2003/03/14 – 2003/07/22
2003/07/09 – 2003/11/12
40
60
80
100
120
140
160
180
200
220
240
260
280
300

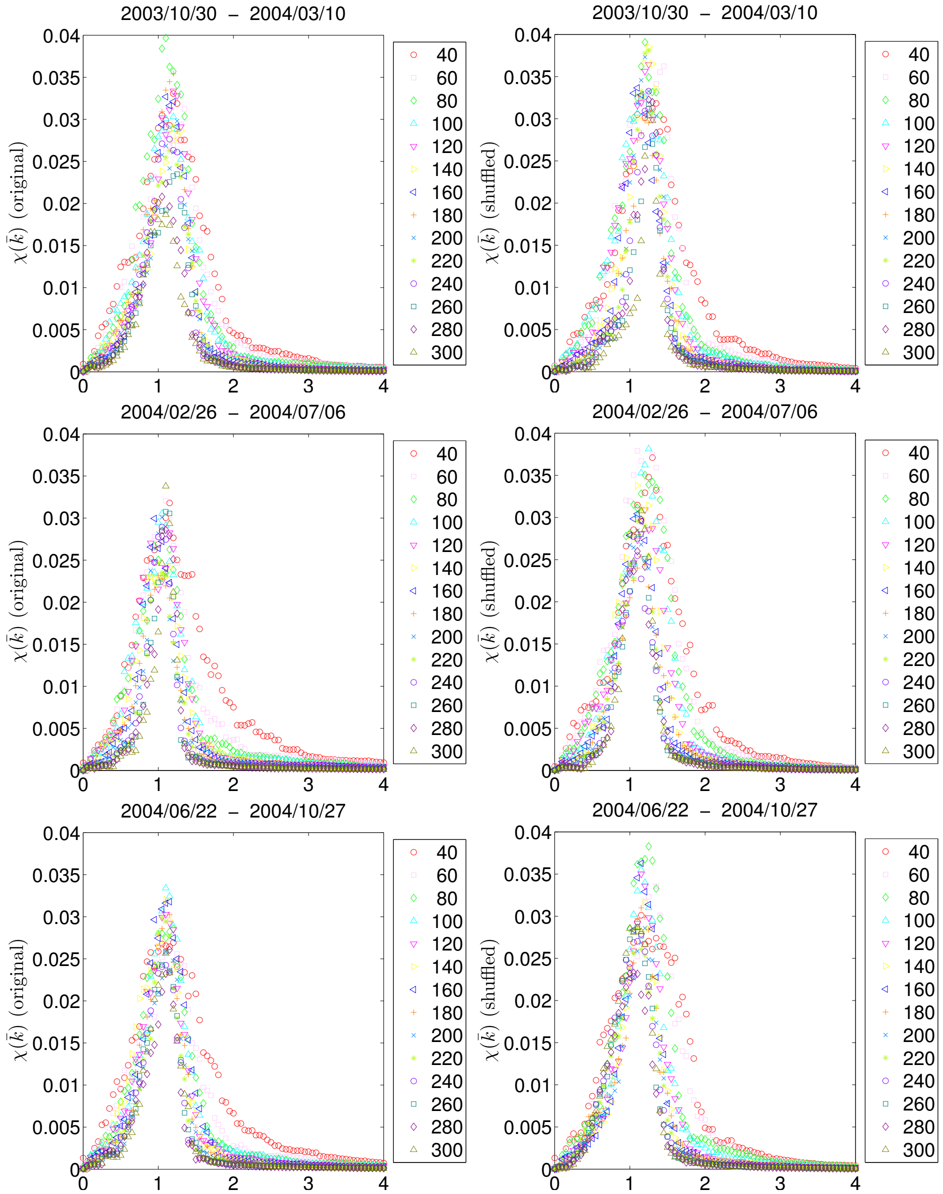
2003/10/30 – 2004/03/10
2004/02/26 – 2004/07/06
2004/06/22 – 2004/10/27
χ(k̄) (original)
χ(k̄) (shuffled)
40
60
80
100
120
140
160
180
200
220
240
260
280
300

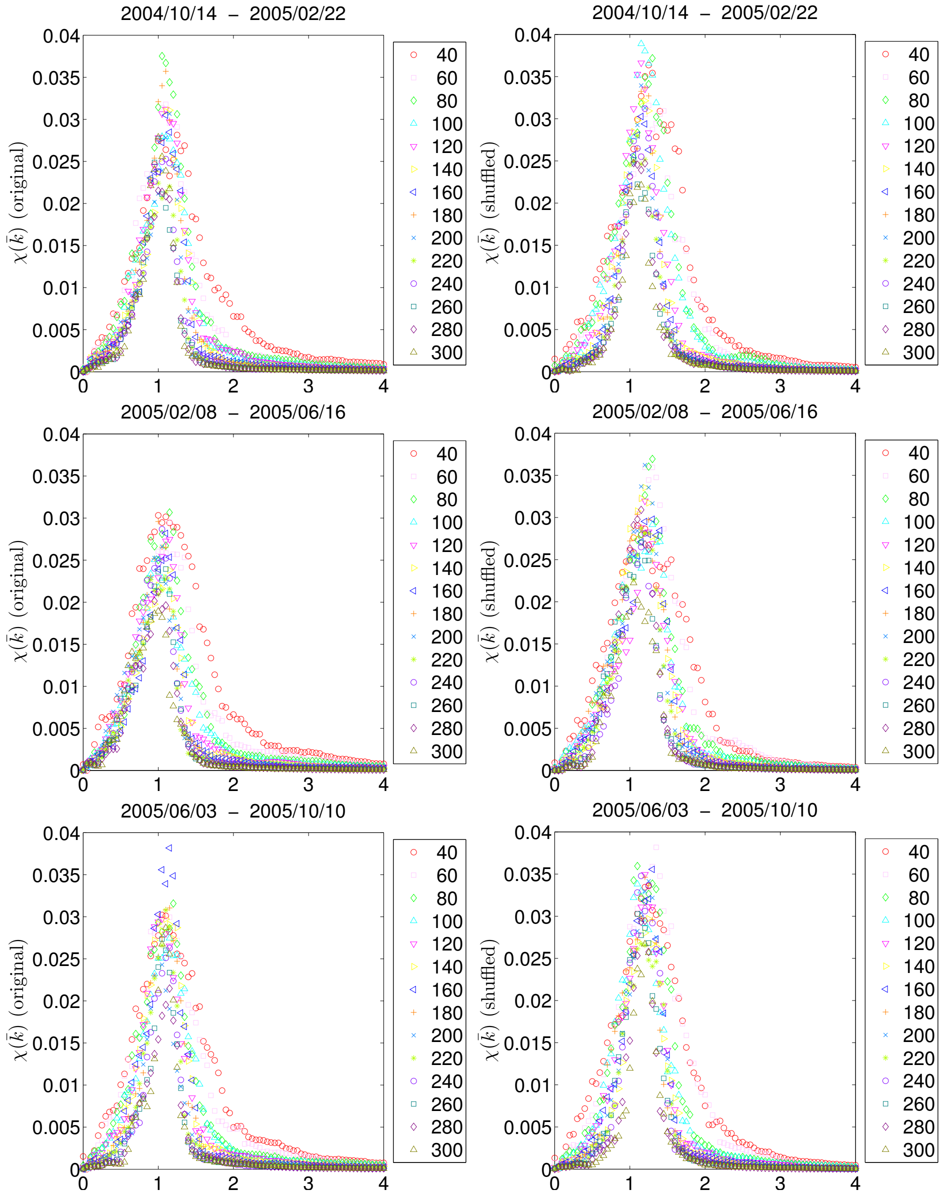
2004/10/14 – 2005/02/22
2005/02/08 – 2005/06/16
2005/06/03 – 2005/10/10
$\chi(\bar{k})$ (original)
$\chi(\bar{k})$ (shuffled)
40
60
80
100
120
140
160
180
200
220
240
260
280
300

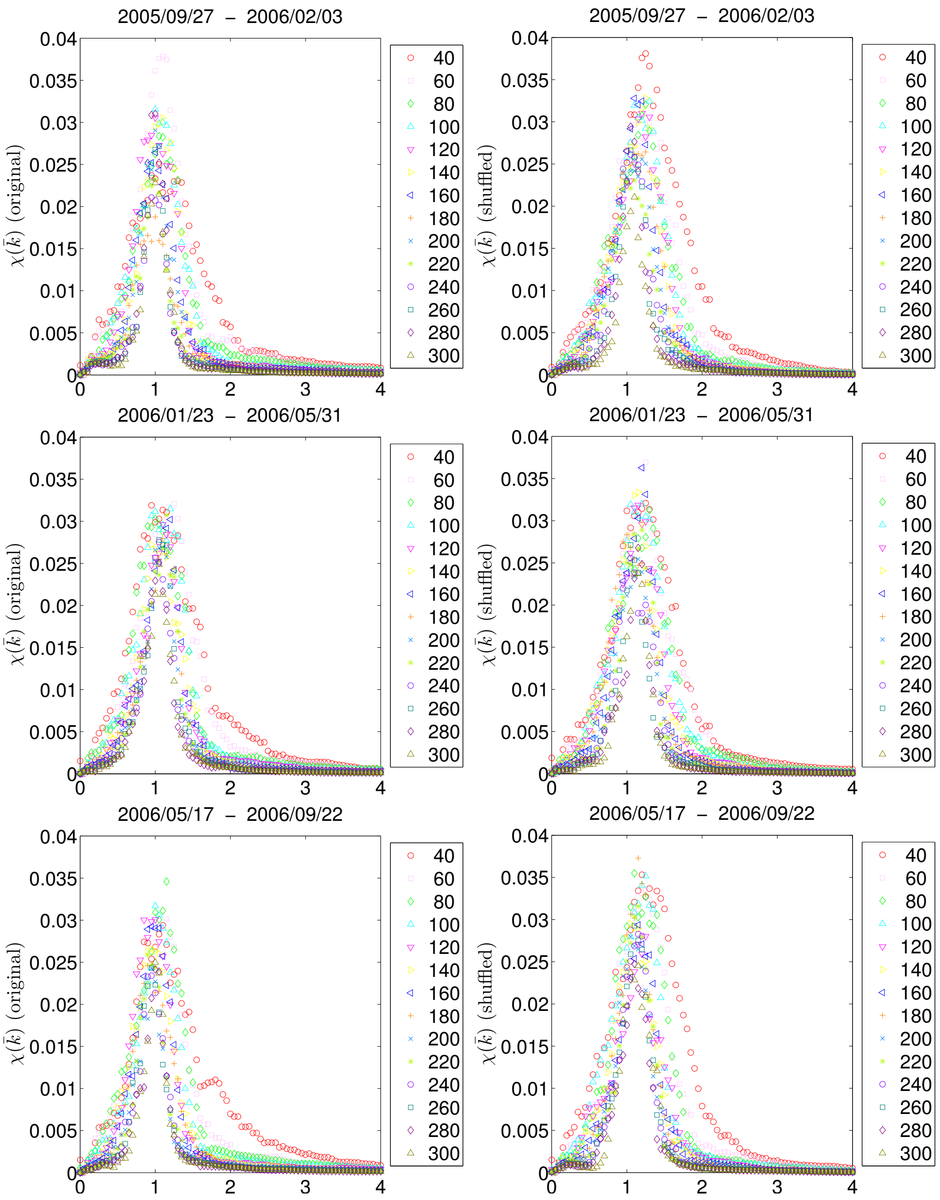

2005/09/27 – 2006/02/03
$\chi(\bar{k})$ (original)
$\chi(\bar{k})$ (shuffled)
2006/01/23 – 2006/05/31
2006/05/17 – 2006/09/22
40
60
80
100
120
140
160
180
200
220
240
260
280
300

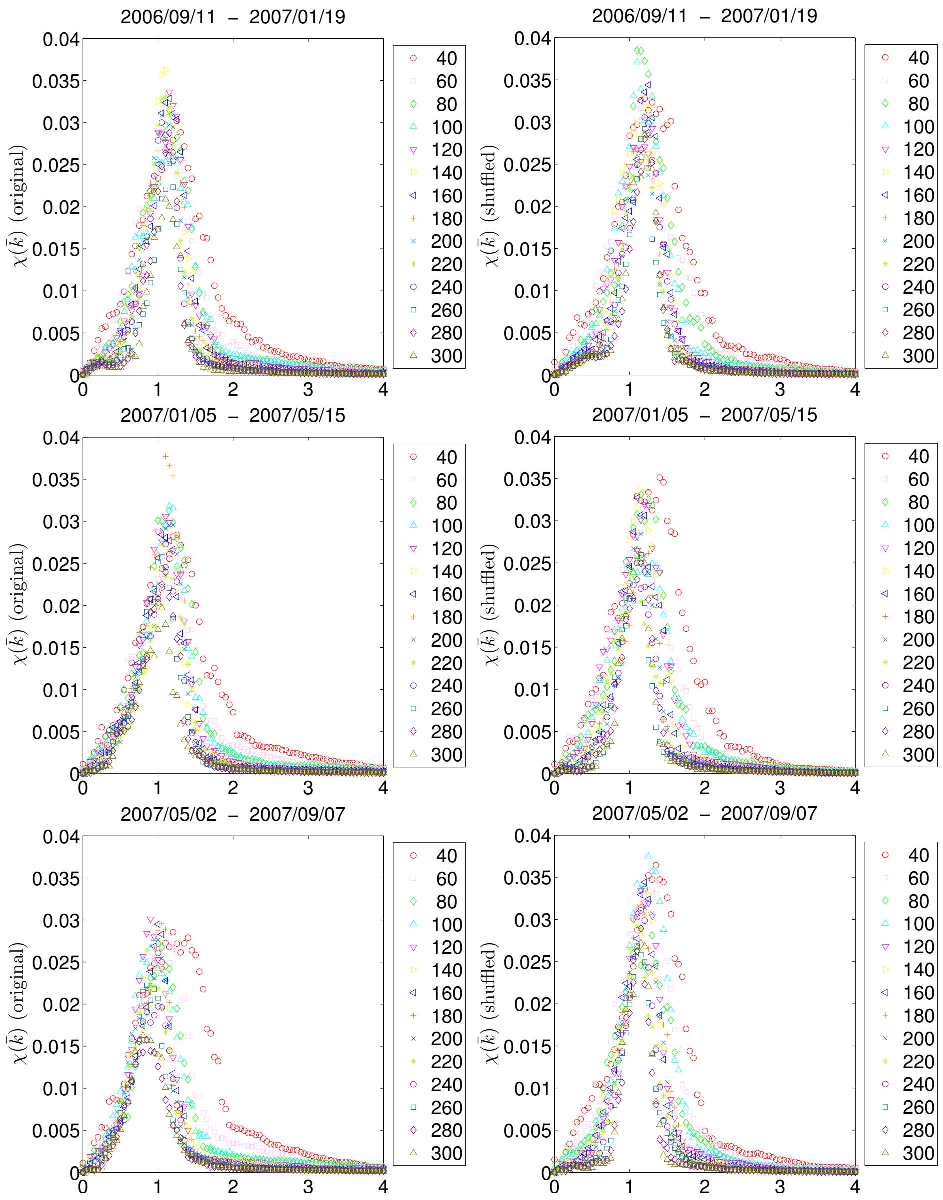

2006/09/11 – 2007/01/19
$\chi(\bar{k})$ (original)
2006/09/11 – 2007/01/19
$\chi(\bar{k})$ (shuffled)
2007/01/05 – 2007/05/15
$\chi(\bar{k})$ (original)
2007/01/05 – 2007/05/15
$\chi(\bar{k})$ (shuffled)
2007/05/02 – 2007/09/07
$\chi(\bar{k})$ (original)
2007/05/02 – 2007/09/07
$\chi(\bar{k})$ (shuffled)
0.04
0.035
0.03
0.025
0.02
0.015
0.01
0.005
0
0
1
2
3
4
40
60
80
100
120
140
160
180
200
220
240
260
280
300

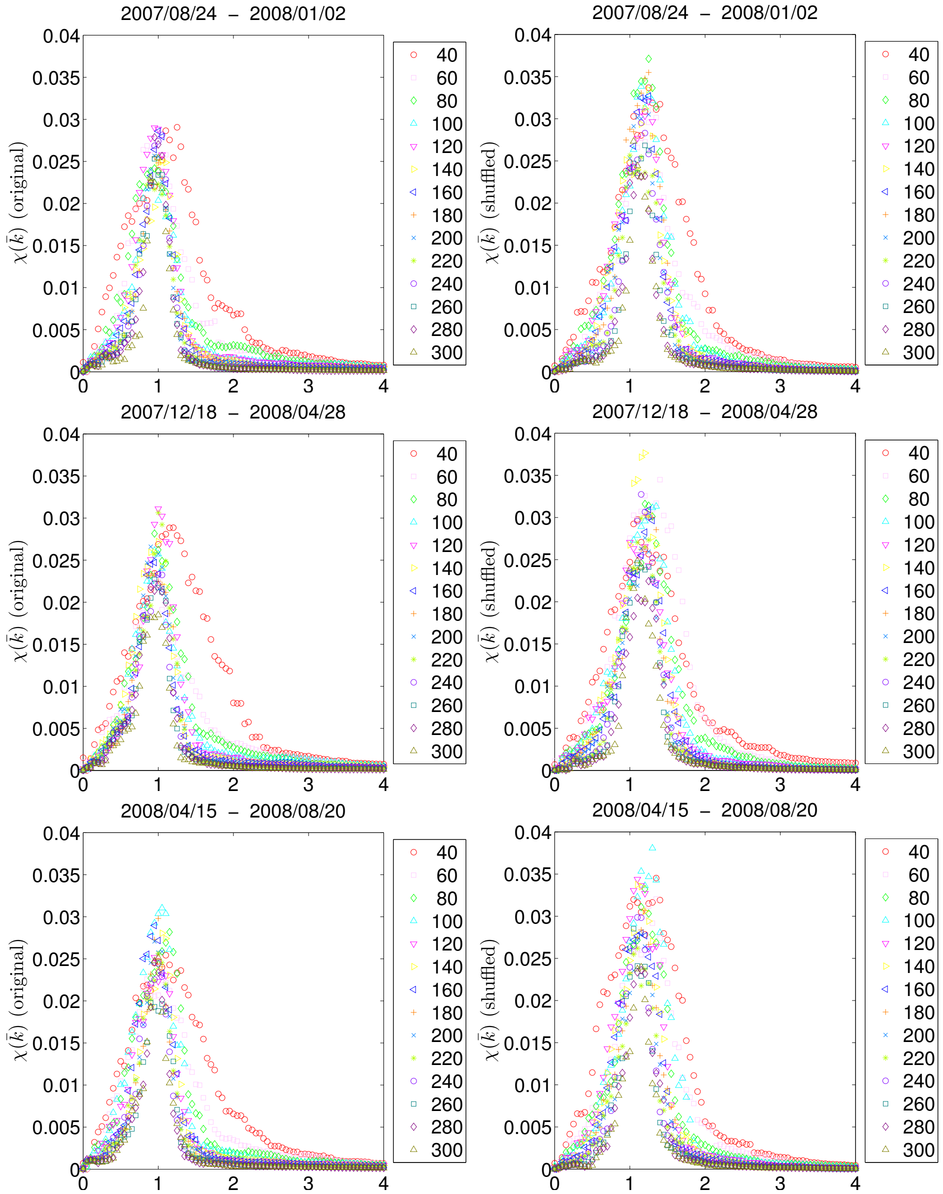

2007/08/24 – 2008/01/02
2007/12/18 – 2008/04/28
2008/04/15 – 2008/08/20
χ(k̄) (original)
χ(k̄) (shuffled)
40
60
80
100
120
140
160
180
200
220
240
260
280
300

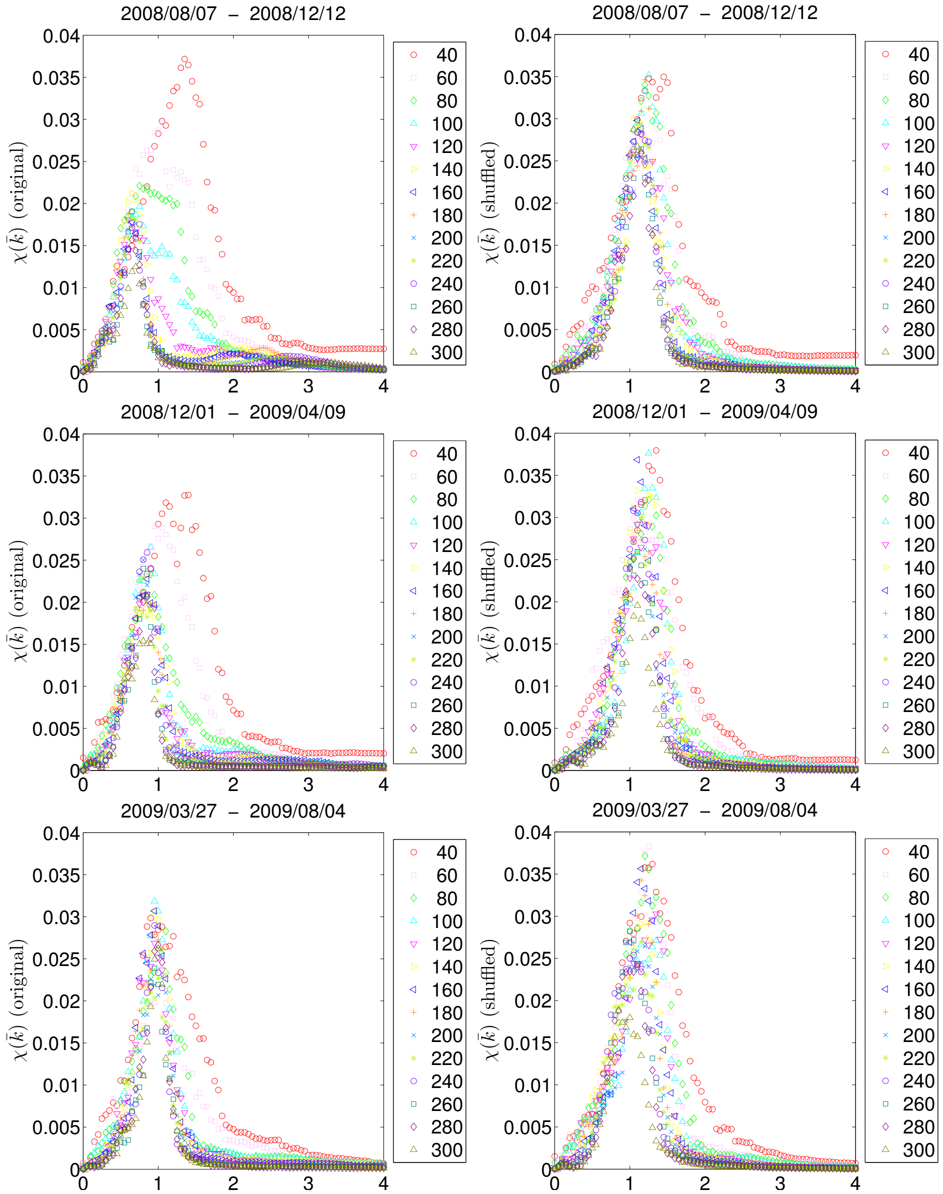
2008/08/07 – 2008/12/12
2008/12/01 – 2009/04/09
2009/03/27 – 2009/08/04
$\chi(\bar{k})$ (original)
$\chi(\bar{k})$ (shuffled)
0.04
0.035
0.03
0.025
0.02
0.015
0.01
0.005
0
0
1
2
3
4
40
60
80
100
120
140
160
180
200
220
240
260
280
300

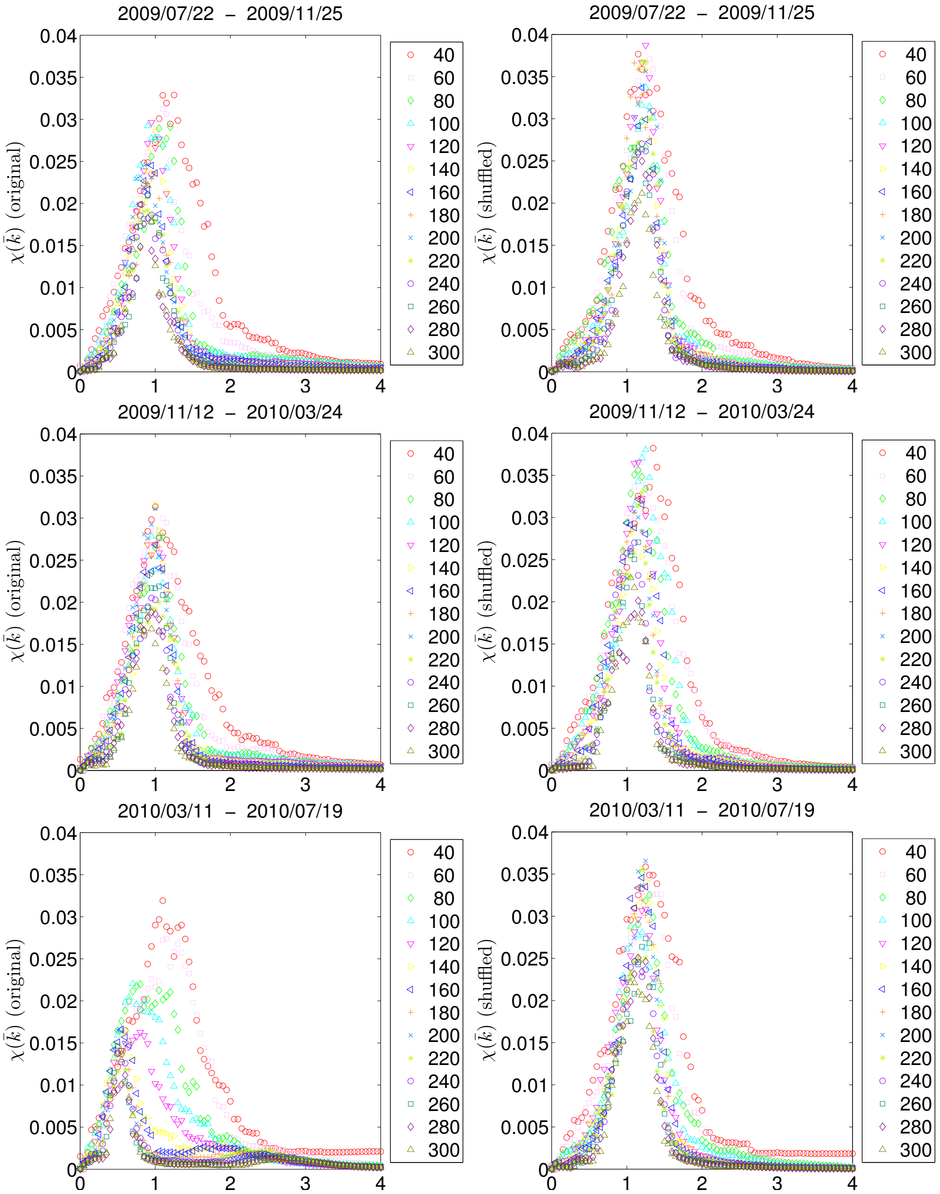
2009/07/22 – 2009/11/25
2009/11/12 – 2010/03/24
2010/03/11 – 2010/07/19
$\chi(\bar{k})$ (original)
$\chi(\bar{k})$ (shuffled)
40
60
80
100
120
140
160
180
200
220
240
260
280
300

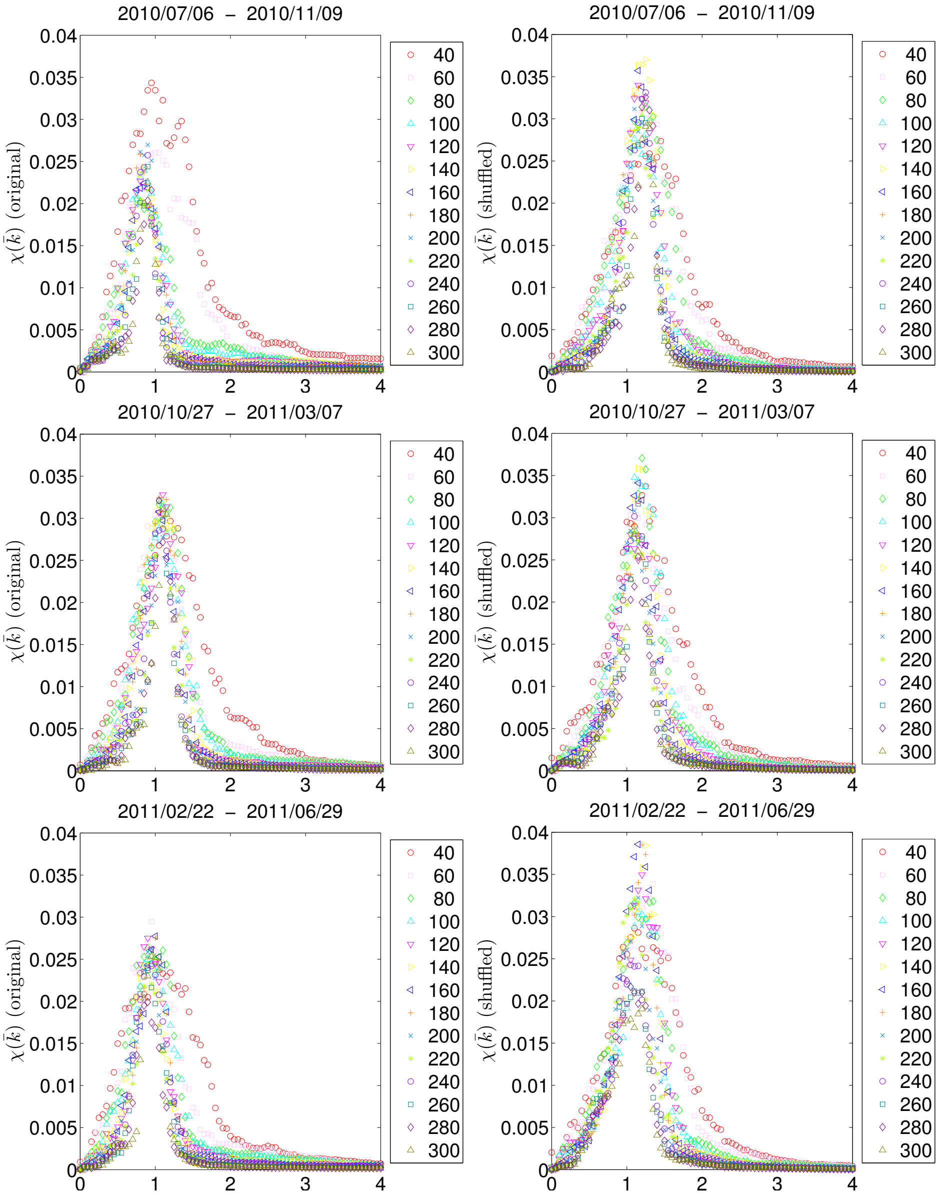
2010/07/06 – 2010/11/09
2010/10/27 – 2011/03/07
2011/02/22 – 2011/06/29
$\chi(\bar{k})$ (original)
$\chi(\bar{k})$ (shuffled)
40
60
80
100
120
140
160
180
200
220
240
260
280
300

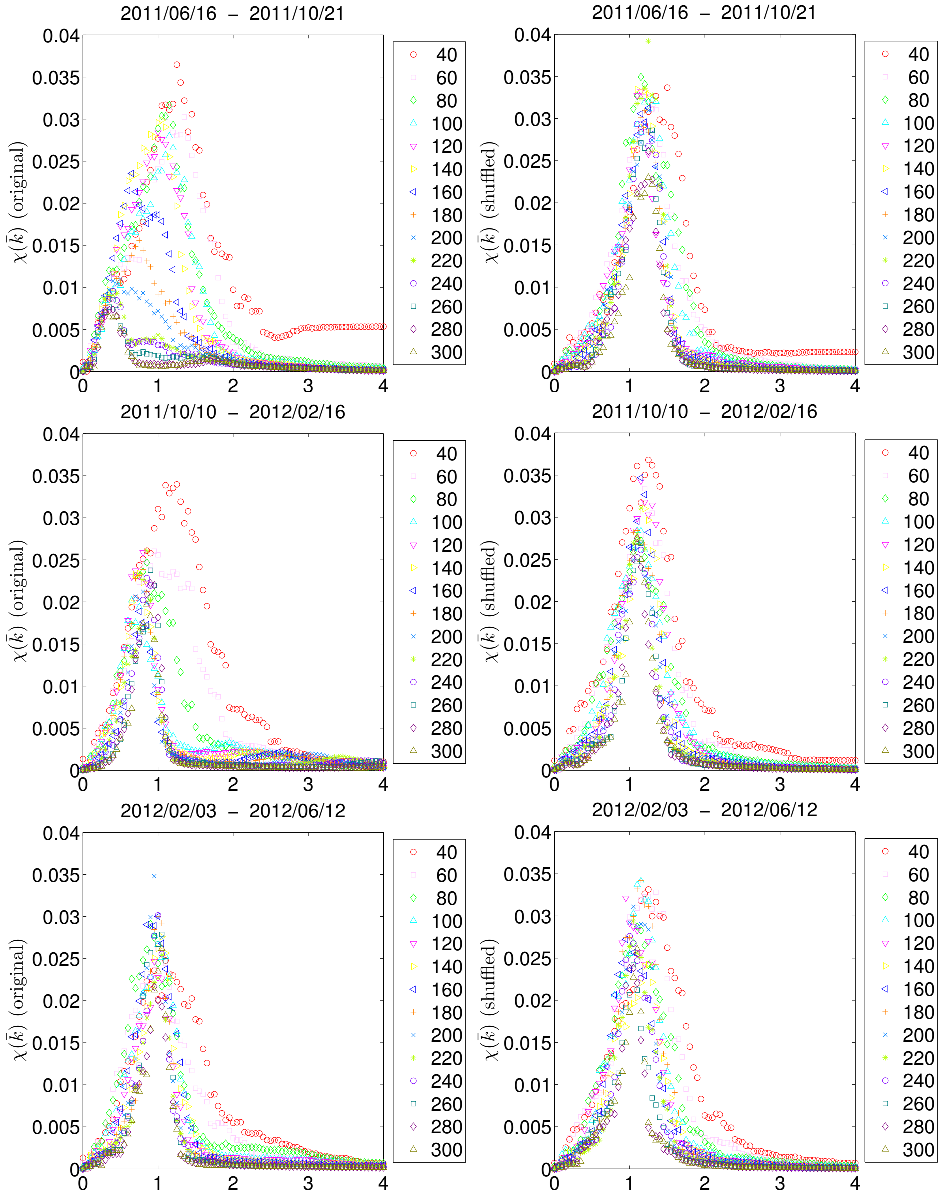

2011/06/16 – 2011/10/21
2011/10/10 – 2012/02/16
2012/02/03 – 2012/06/12
$\chi(\bar{k})$ (original)
$\chi(\bar{k})$ (shuffled)
40
60
80
100
120
140
160
180
200
220
240
260
280
300

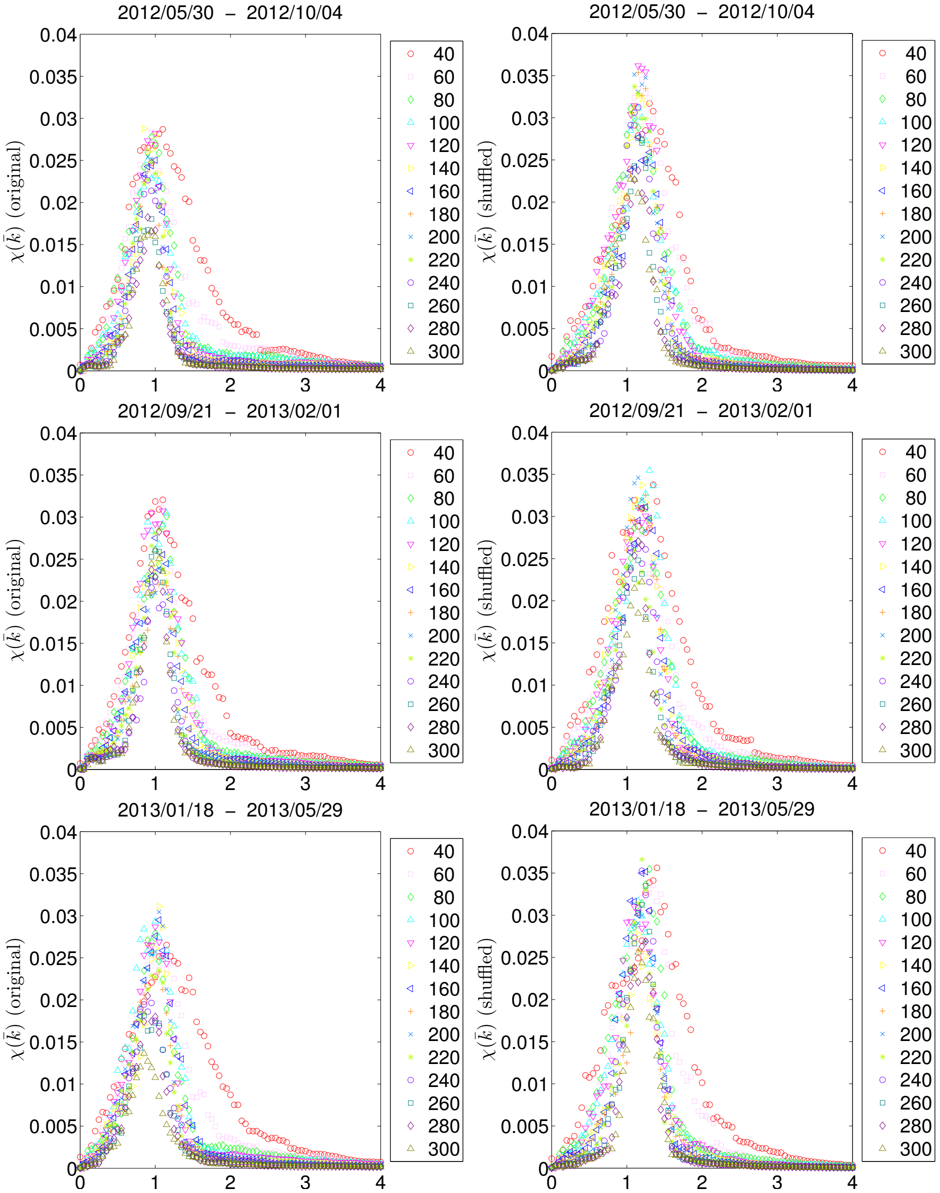

2012/05/30 – 2012/10/04
2012/09/21 – 2013/02/01
2013/01/18 – 2013/05/29
$\chi(\bar{k})$ (original)
$\chi(\bar{k})$ (shuffled)
40
60
80
100
120
140
160
180
200
220
240
260
280
300

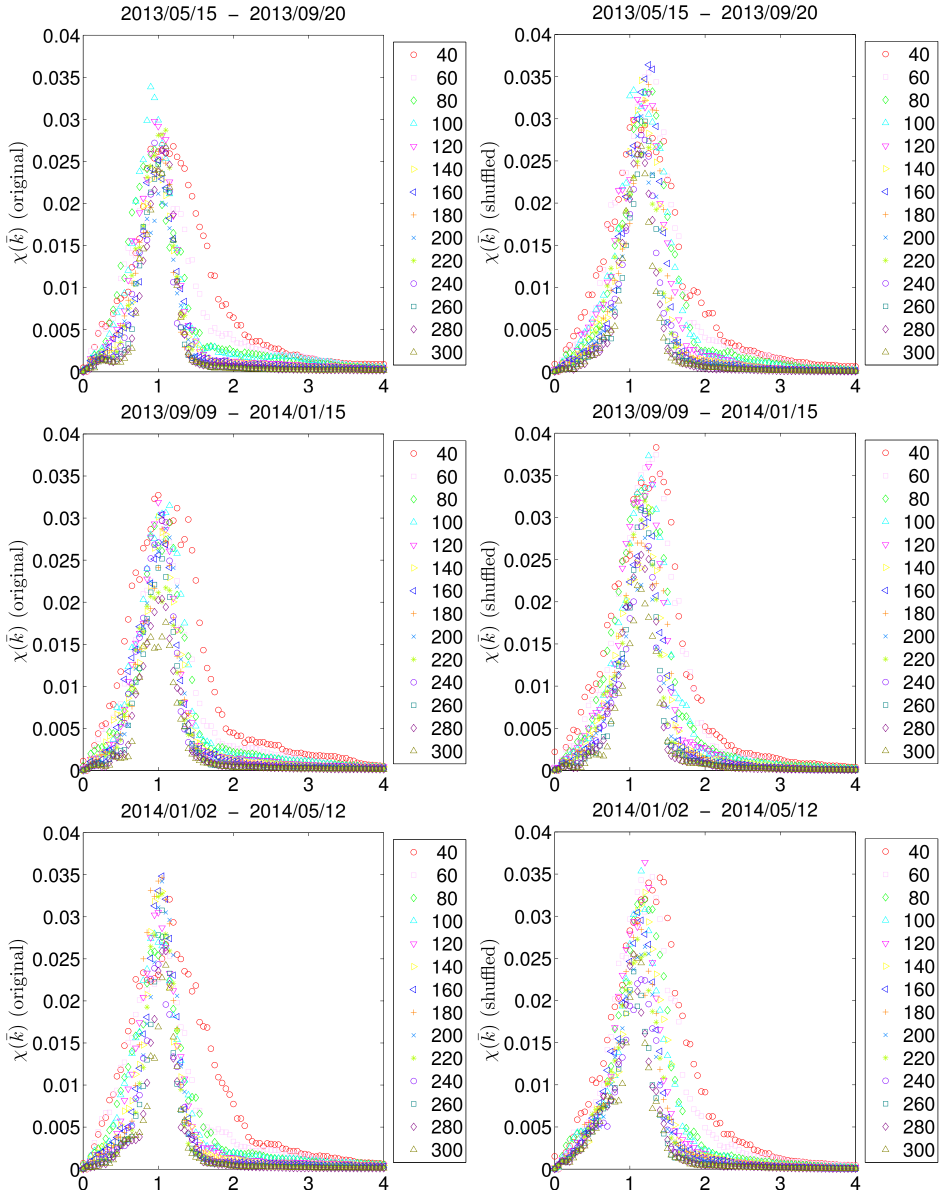
2013/05/15 – 2013/09/20
2013/09/09 – 2014/01/15
2014/01/02 – 2014/05/12
χ(k̄) (original)
χ(k̄) (shuffled)
0
0.005
0.01
0.015
0.02
0.025
0.03
0.035
0.04
0
1
2
3
4
40
60
80
100
120
140
160
180
200
220
240
260
280
300

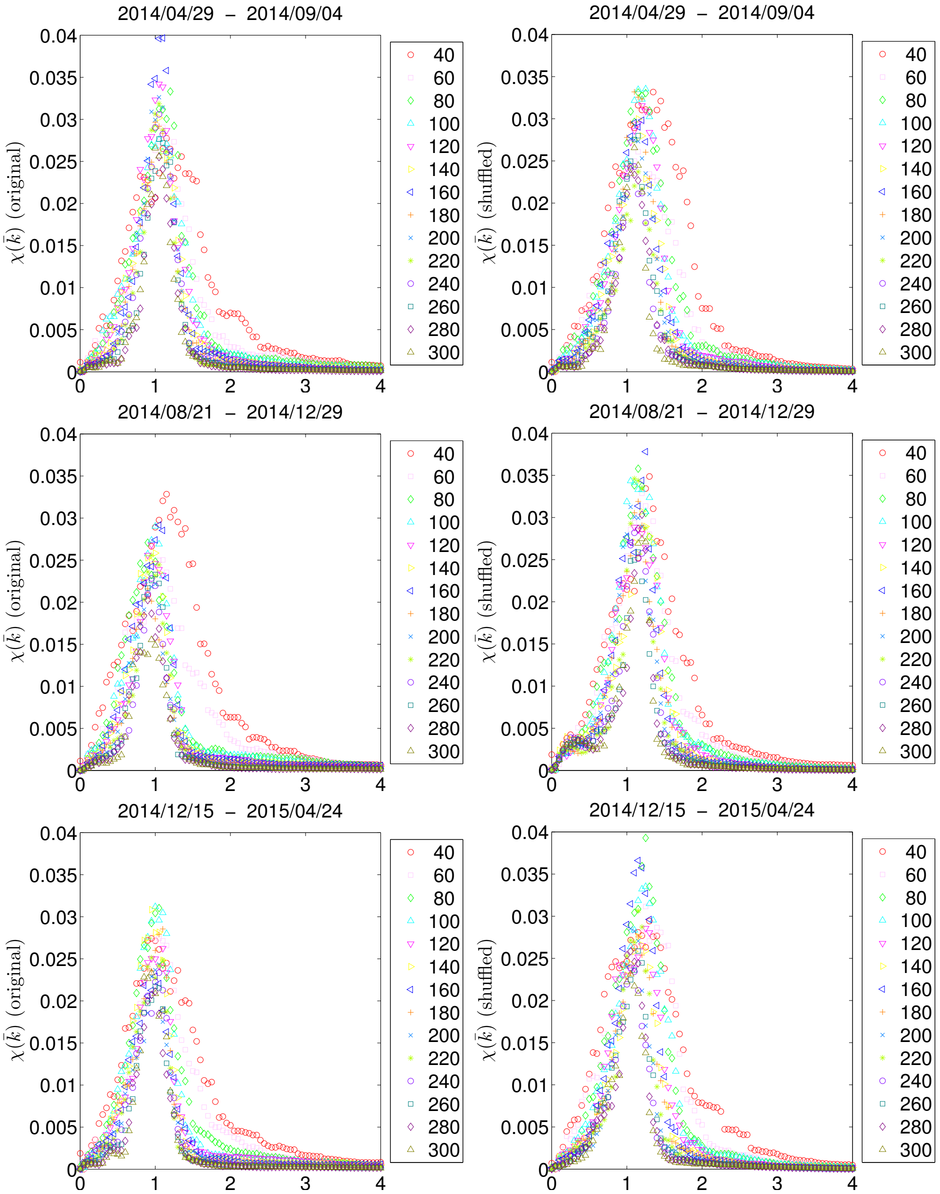

2014/04/29 – 2014/09/04
2014/08/21 – 2014/12/29
2014/12/15 – 2015/04/24
$\chi(\bar{k})$ (original)
$\chi(\bar{k})$ (shuffled)
0
0.005
0.01
0.015
0.02
0.025
0.03
0.035
0.04
1
2
3
4
40
60
80
100
120
140
160
180
200
220
240
260
280
300

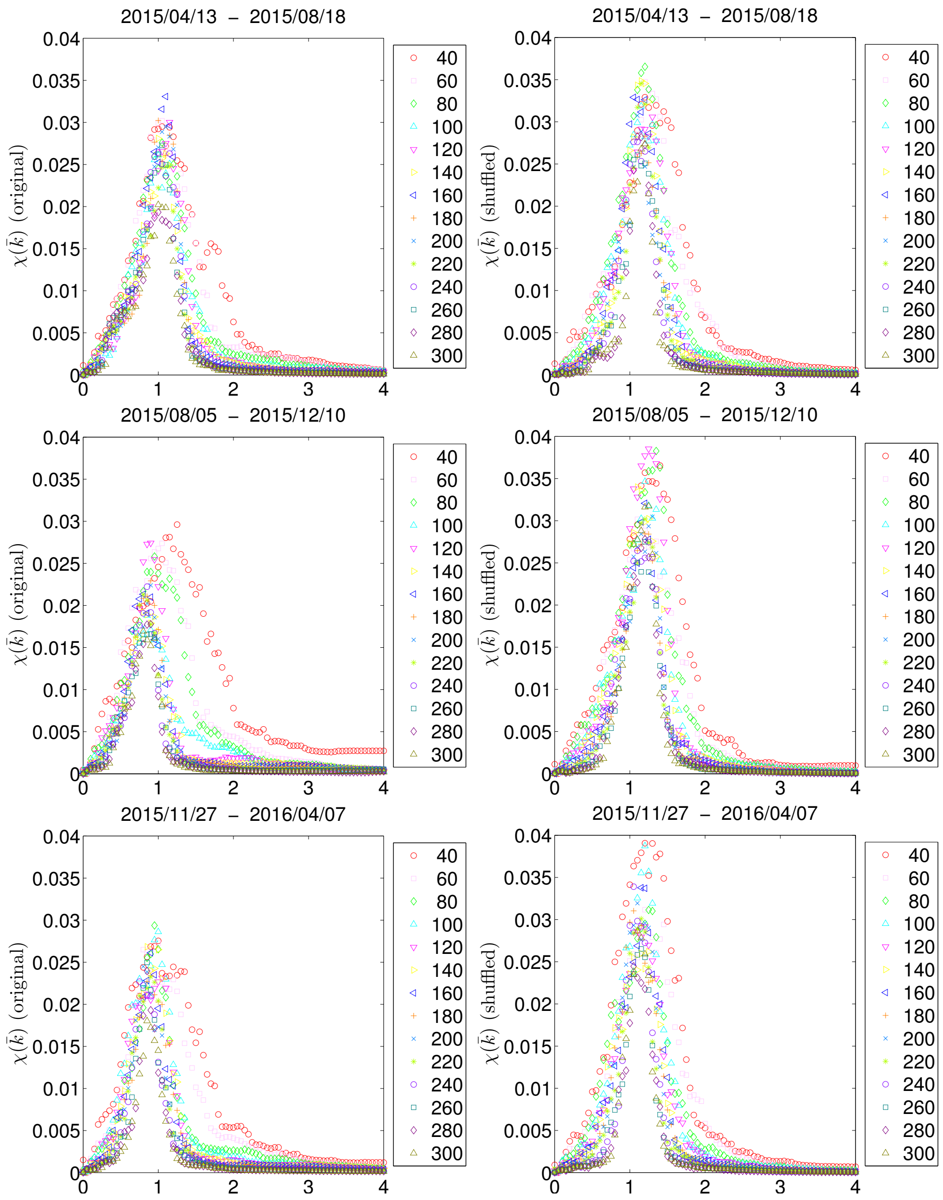
2015/04/13 – 2015/08/18
2015/08/05 – 2015/12/10
2015/11/27 – 2016/04/07
χ(k̄) (original)
χ(k̄) (shuffled)
0 0.005 0.01 0.015 0.02 0.025 0.03 0.035 0.04
0 1 2 3 4
40 60 80 100 120 140 160 180 200 220 240 260 280 300

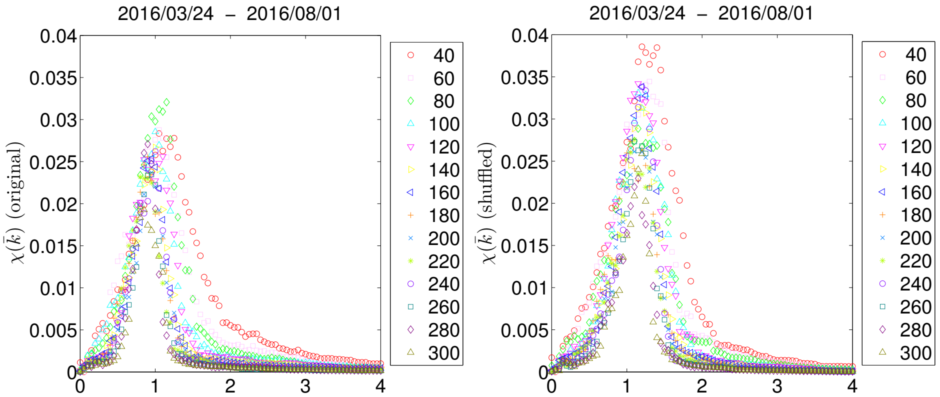

2016/03/24 – 2016/08/01
χ(k̄) (original)
2016/03/24 – 2016/08/01
χ(k̄) (shuffled)
40
60
80
100
120
140
160
180
200
220
240
260
280
300

Calculations

We have checked giant component probability as a function of threshold level, instead of mean degree. Main reason that we used mean degree was the different sub-graph sizes. Critical point of threshold level will be changed in different sub-graph sizes, in comparison to mean degree which is fixed (Fig. 1, 2).

Another situation we have checked was working with correlation matrix, instead of interaction matrix. Fig. 3 shows the giant component probability of correlation network , similar to the process mentioned in the article. The sharpness of critical behavior disappears here, probably due to the non-local interactions propagated in the network's weights. Also responses to the different times are less prominent.

Also to compare our results with ER random network theory, we calculate giant component probability of a random network[1,2]. Suppose $P_{\infty}^{theo.}(\bar{k})$ is the probability that a node is connected to giant component, in random network with size N and mean degree $\bar{k}$. Then, this is the complementary probability that a node is not connected to giant component, considering other nodes:

$$1 - P_{\infty}^{theo.}(\bar{k}) = \left(\left(1 - \frac{\bar{k}}{N-1}\right) + \frac{\bar{k}}{N-1}\left(1 - B^{theo}(N,\bar{k})\right)\right)^{N-1} = \left(1 - \frac{\bar{k}}{N-1} P_{\infty}^{theo.}(\bar{k})\right)^{N-1} \quad (1)$$

It means a node i is not connected to another node j or is connected to j, but j is not connected to giant component. In the limit of large N and finite $\bar{k}$, the equation could be written as follow:

$$P_{\infty}^{theo.}(\bar{k}) = 1 - e^{-\bar{k}P_{\infty}^{theo.}(\bar{k})} \quad (2)$$

which $P_{\infty}^{theo.}(\bar{k})$ is the giant component probability of a ER random network. Solving this self-consistent equation yields the giant component probability as a function of mean degree in a ER random network. Now we can compare giant component probability in real networks with theory prediction. Fig. 4 shows the absolute difference between a giant component probability and the theory, as a function of sub-graph size and the time period of the network.

For a better demonstration, we find the maximum point of susceptibility in each curve and call its corresponding mean degree $\bar{k}(G_\theta(t,s))$ as the critical point. Fig. 5-8(a,b) depicts the critical points $\bar{k}_c(t,s)$ and their corresponding threshold level θ_c, for time window length 30, 60, 90 and 120 working days. Also, as an another measure to evaluate the criticality of each transition, we calculate deviation of susceptibility curves from its maximum as follows:

$$dev^2(s) = \left\langle \chi^2(\bar{k}(G_\theta(t,s))) \left(\bar{k}(G_\theta(t,s)) - \bar{k}_c(t,s)\right)^2 \right\rangle_\theta \quad (3)$$

Fig. 5-8(c) depicts the square root of this variable over the time. It shows that although critical points shift to lesser degrees (and consequently in higher threshold), criticality demolishes close to financial crisis.

Fig. **??** depicts the difference between giant component and its theory prediction (like Fig. 5 of the main paper) for 30, 60, 90 and 120 working days.

References

1. M. E. J. Newman, *Networks: An introduction*, (Chapter 12, Oxford University Press, 2010).
2. A. L. Barabasi, *Network Science*, Chapter 3, barabasi.com/networksciencebook/.

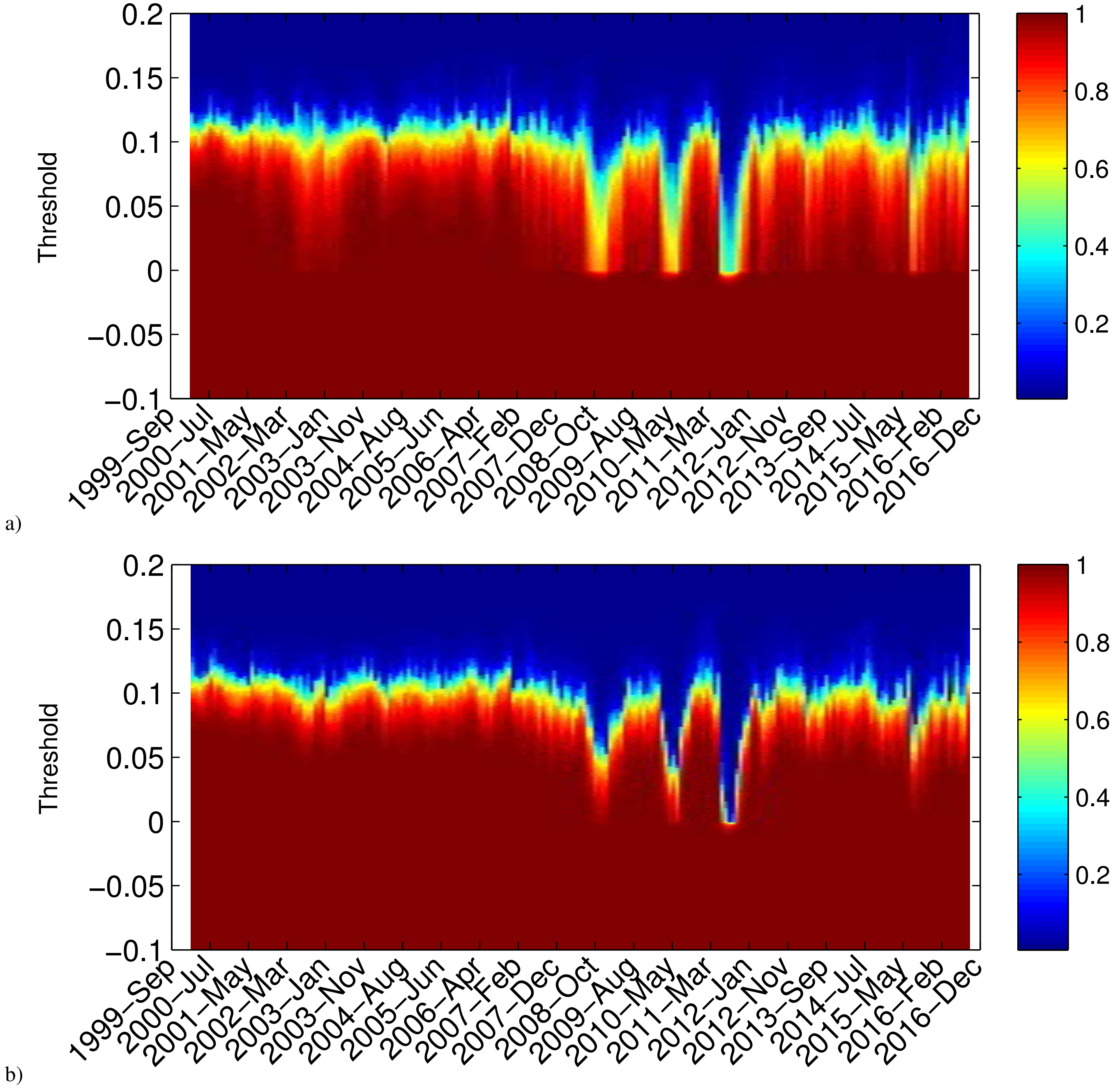

Figure 1. Giant component probability in whole network, as a function of threshold level (vertical axis) and through the time (horizontal axis). a) original networks and b) their shuffled network. Time window length is 90.

a) b)

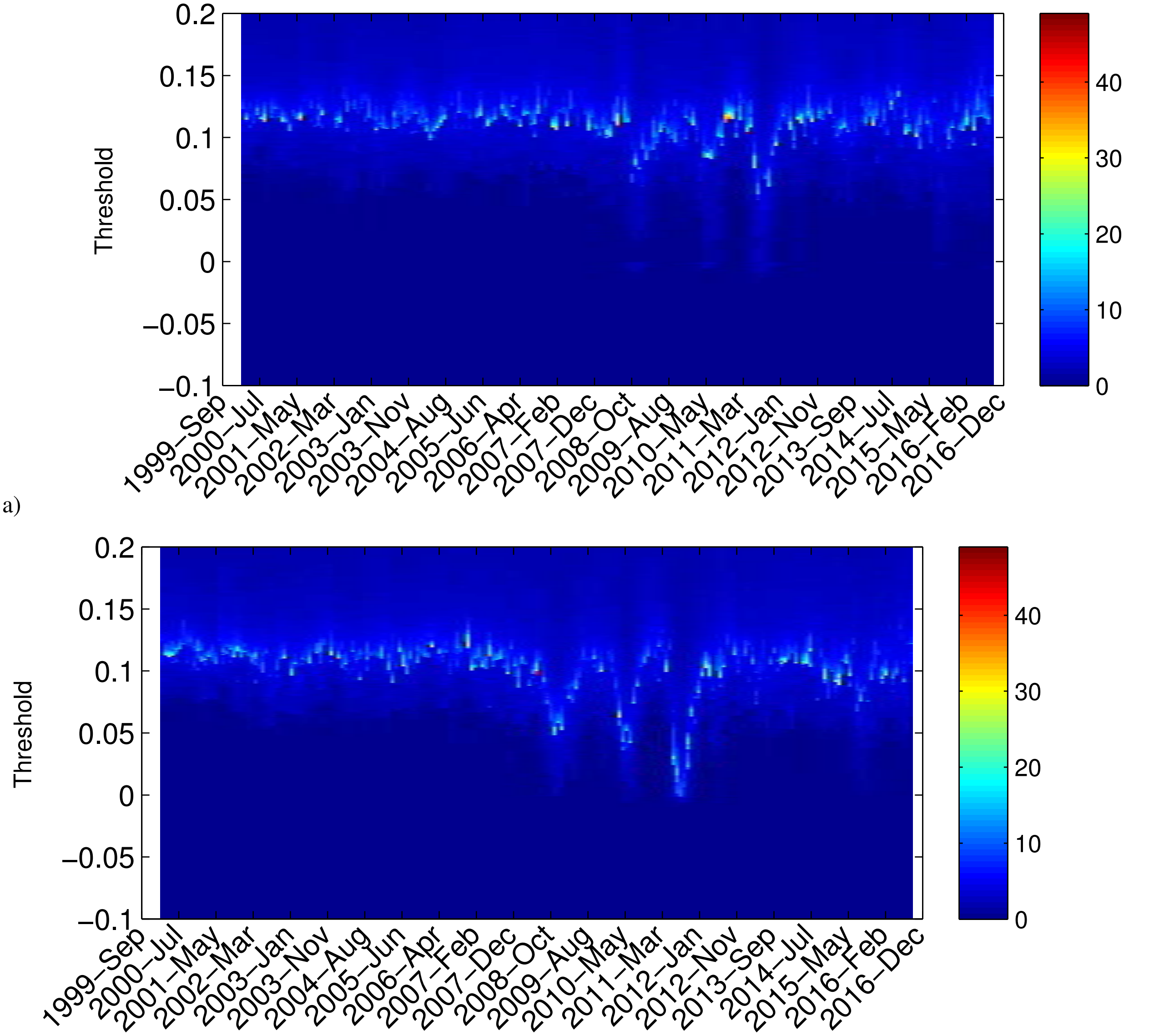

Figure 2. Mean cluster size in whole network, as a function of threshold level (vertical axis) and through the time (horizontal axis). a) original networks and b) their shuffled network.

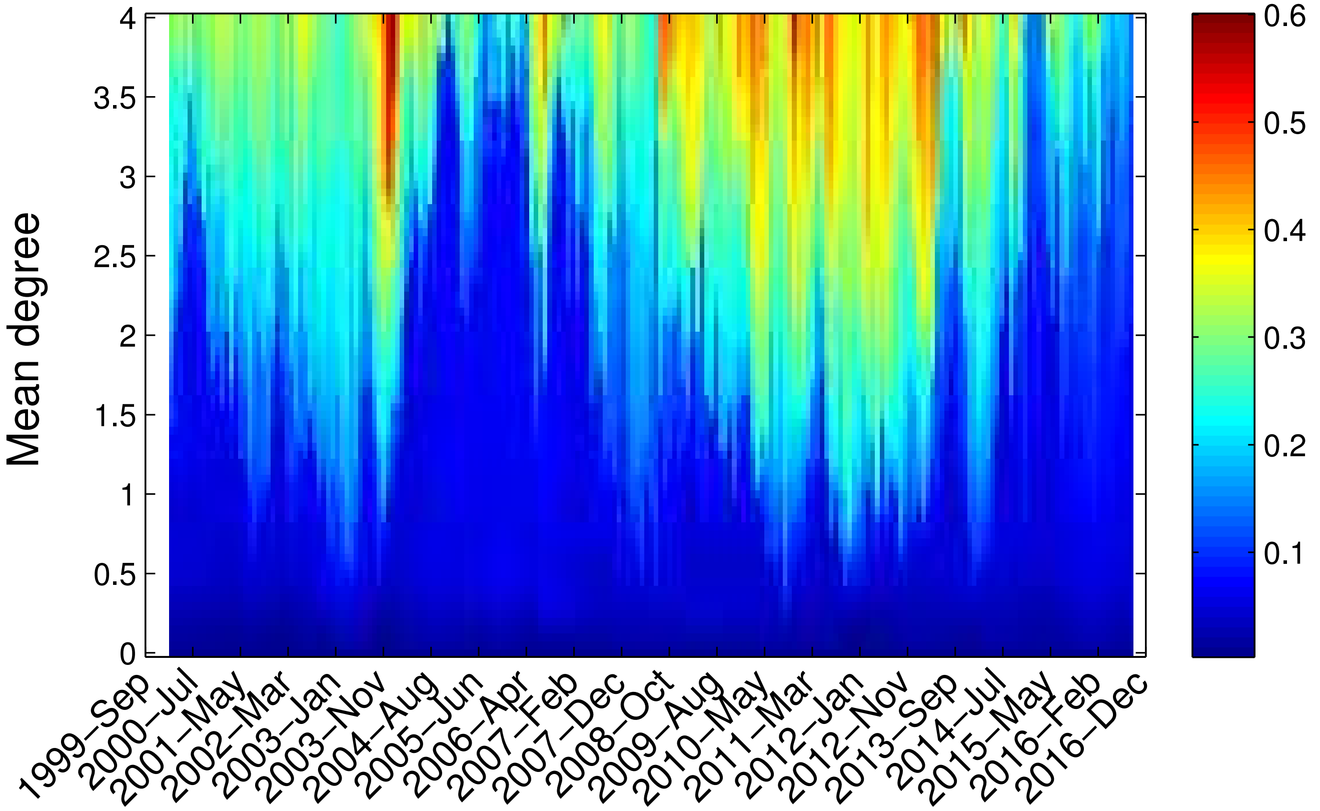

a)

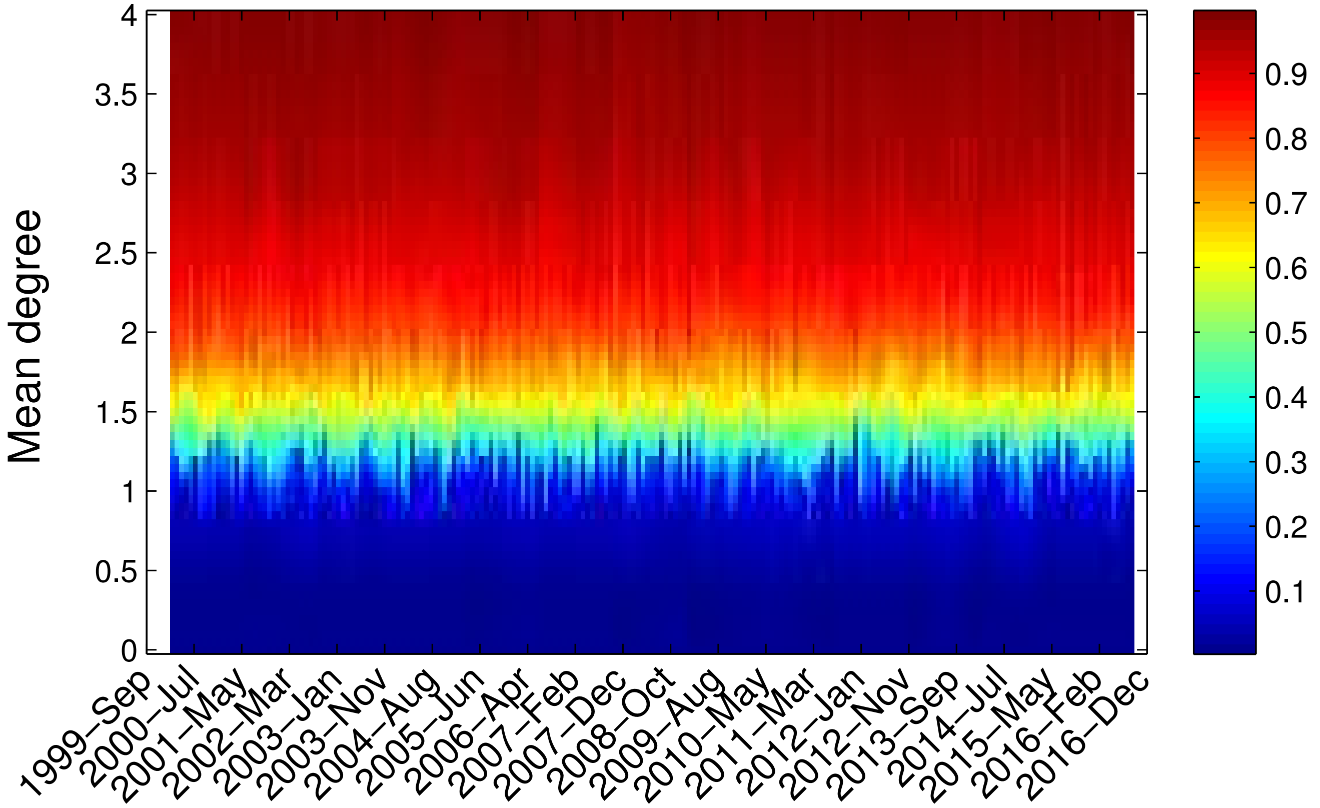

b)

Figure 3. Giant component probability of correlation network (in whole network), as a function of mean degree (vertical axis) and through the time (horizontal axis). a) original networks and b) their shuffled network. Time window length is 90.

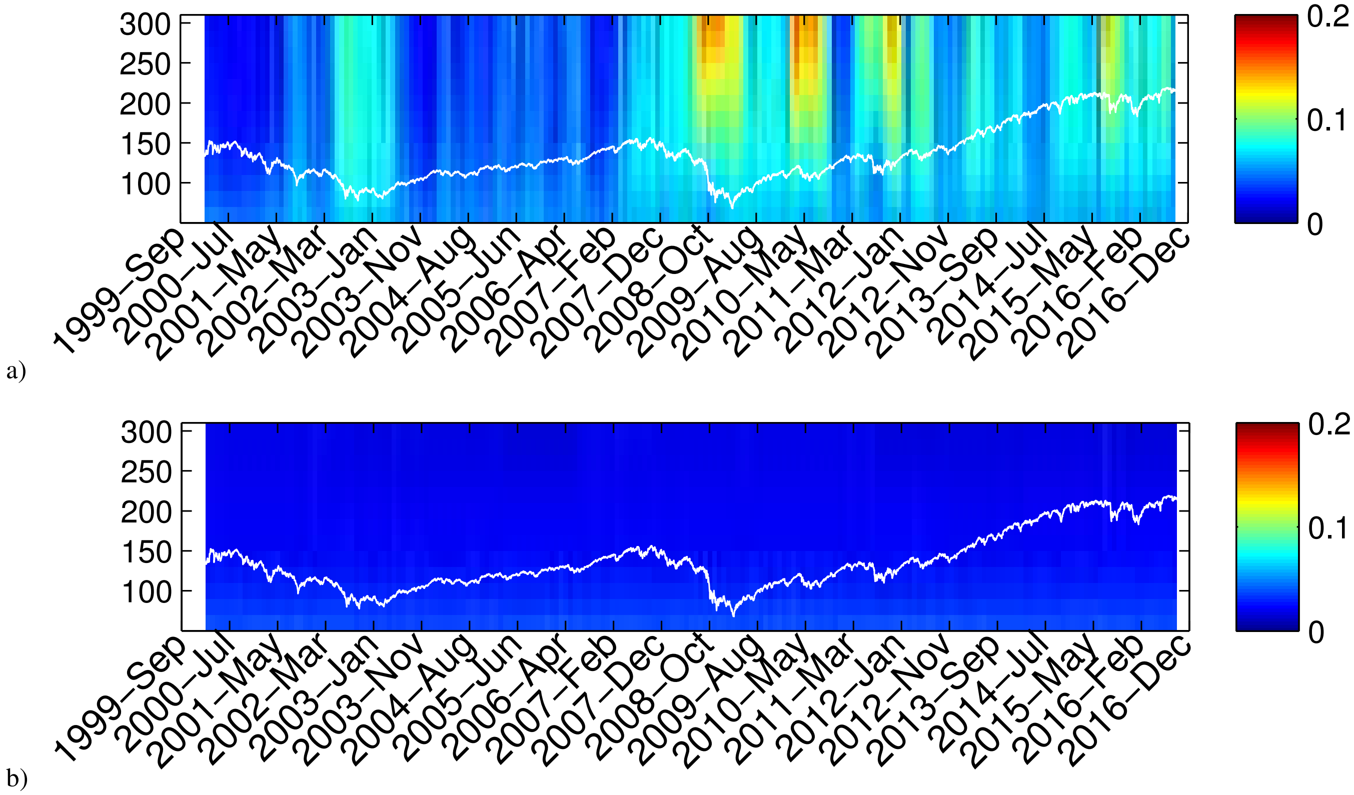

Figure 4. Absolute difference between giant component probability and the theory prediction, as a function of sub-graph size (vertical axis) and through the time (horizontal axis). a) original networks and b) their shuffled network Time window length is 90.

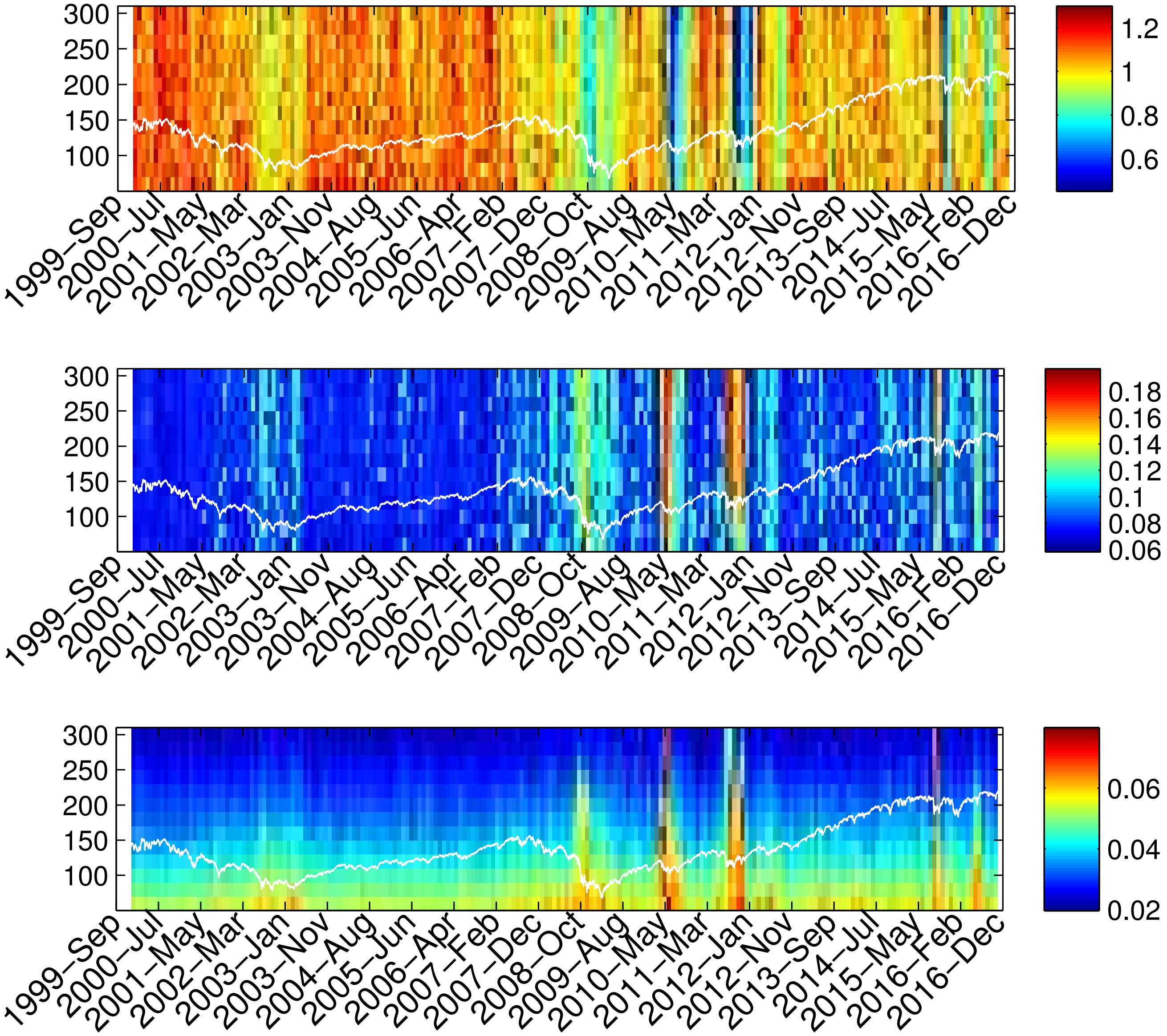

a)

b)

c)

Figure 5. Critical point (maximum points of susceptibility curves) as a) mean degree and b) threshold level. c) deviation of susceptibility curves from its maximum. Horizontal dimension corresponds to different time periods (with time window = 30 working days) and vertical dimension corresponds to different sub-sample sizes. S&P500 index is scaled and plotted (with white color) as a indicator of financial crises.

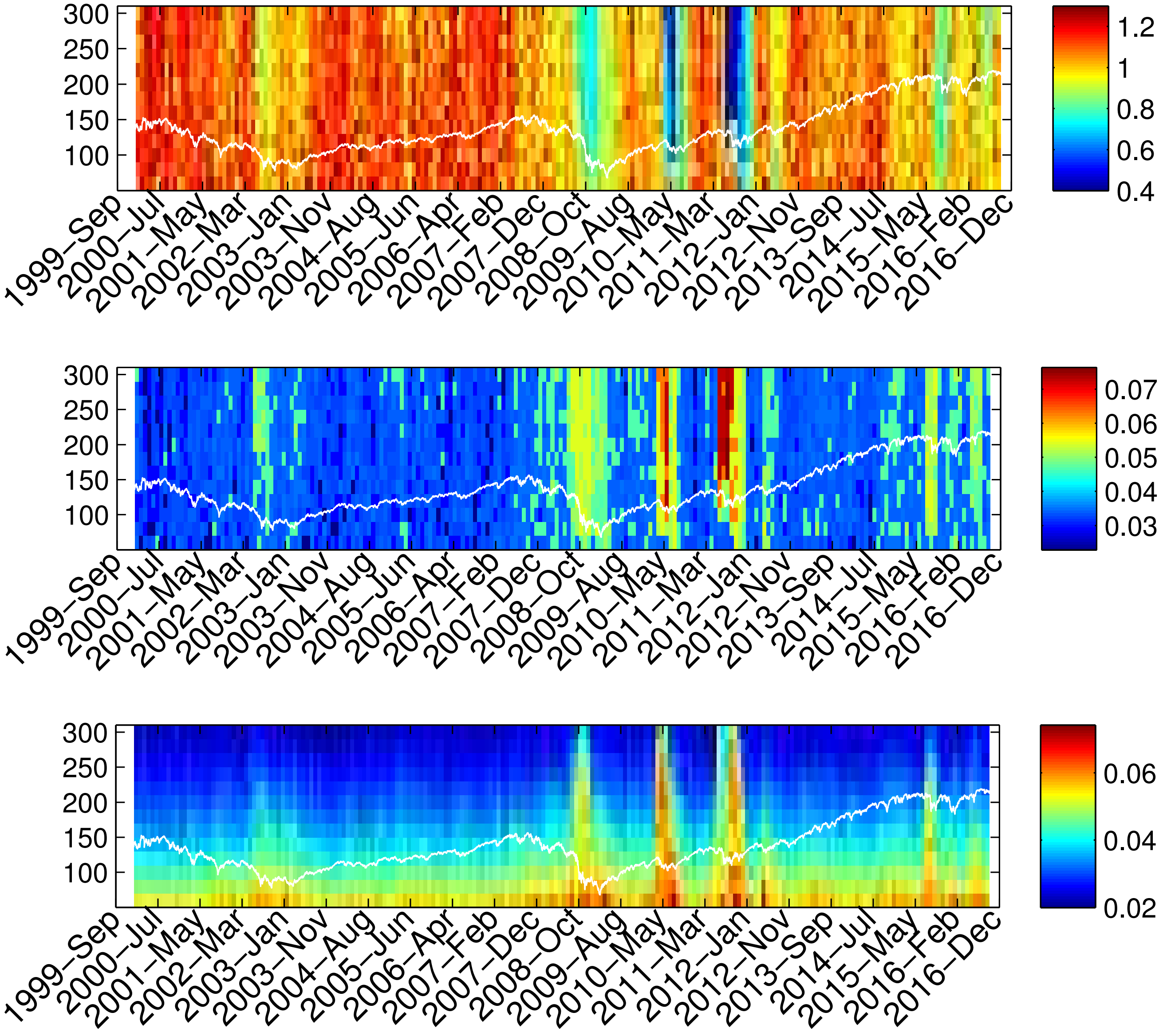

a)

b)

c)

Figure 6. Critical point (maximum points of susceptibility curves) as a) mean degree and b) threshold level. c) deviation of susceptibility curves from its maximum. Horizontal dimension corresponds to different time periods (with time window = 60 working days) and vertical dimension corresponds to different sub-sample sizes. S&P500 index is scaled and plotted (with white color) as a indicator of financial crises.

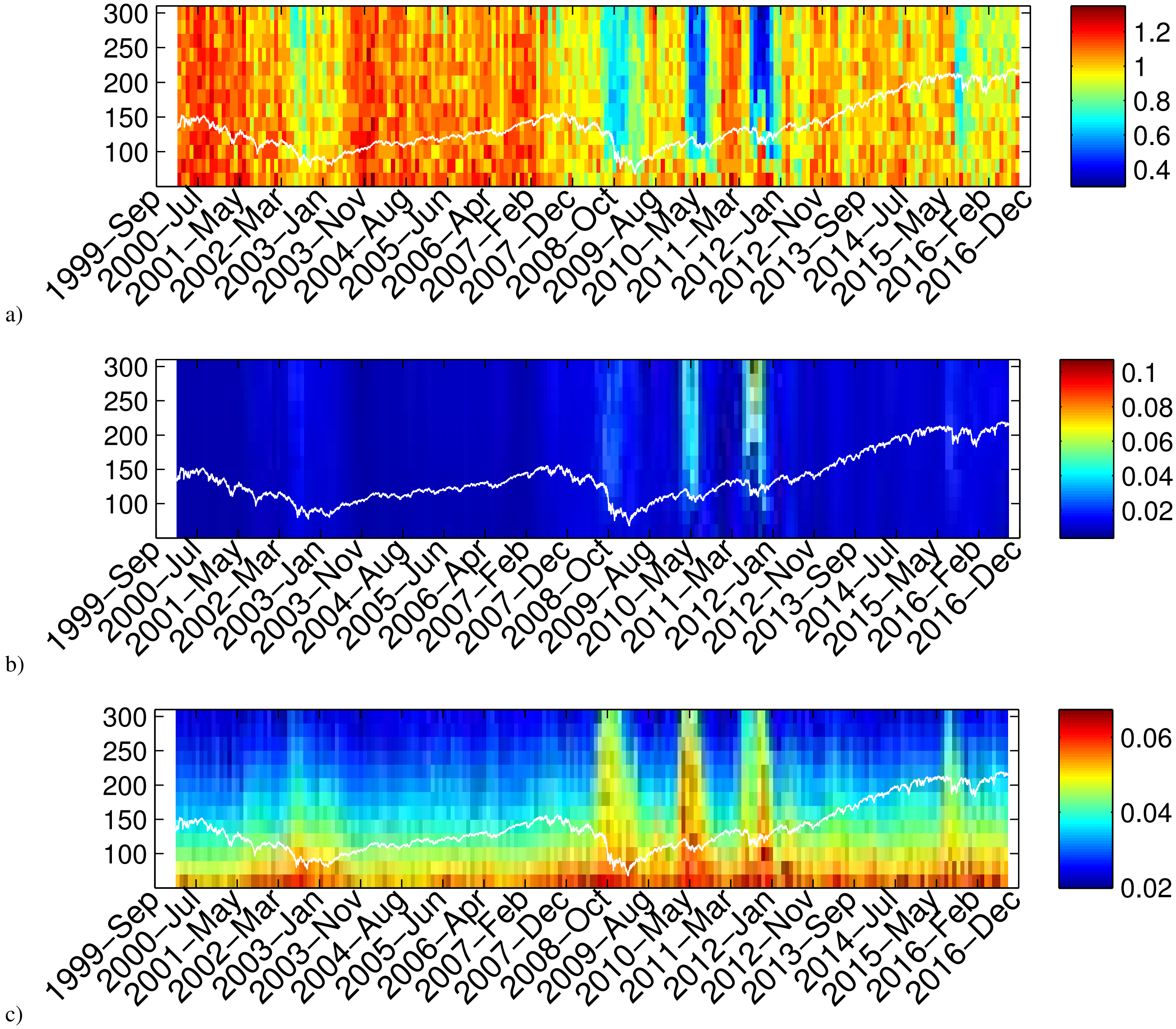

Figure 7. Critical point (maximum points of susceptibility curves) as a) mean degree and b) threshold level. c) deviation of susceptibility curves from its maximum. Horizontal dimension corresponds to different time periods (with time window = 90 working days) and vertical dimension corresponds to different sub-sample sizes. S&P500 index is scaled and plotted (with white color) as a indicator of financial crises.

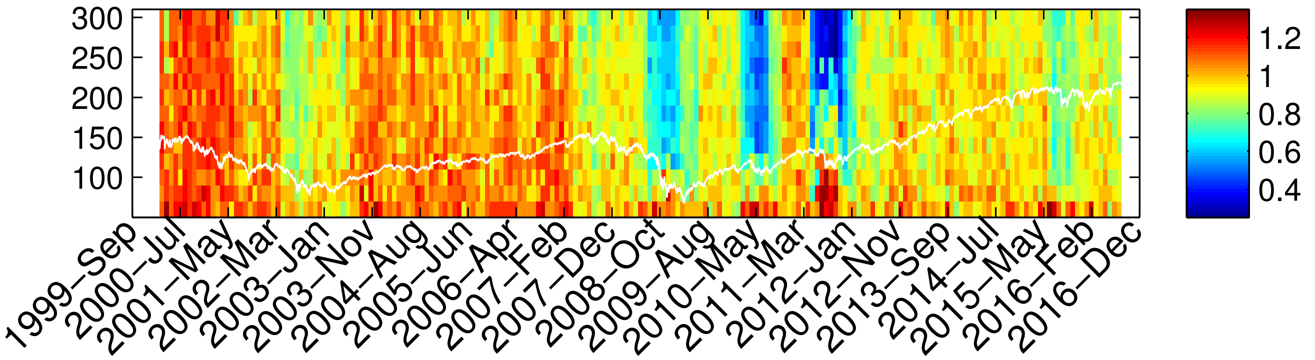

a)

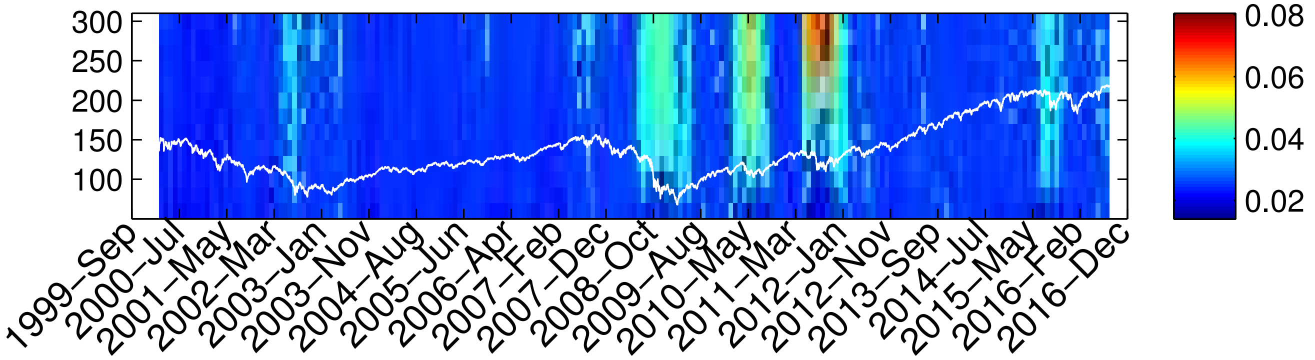

b)

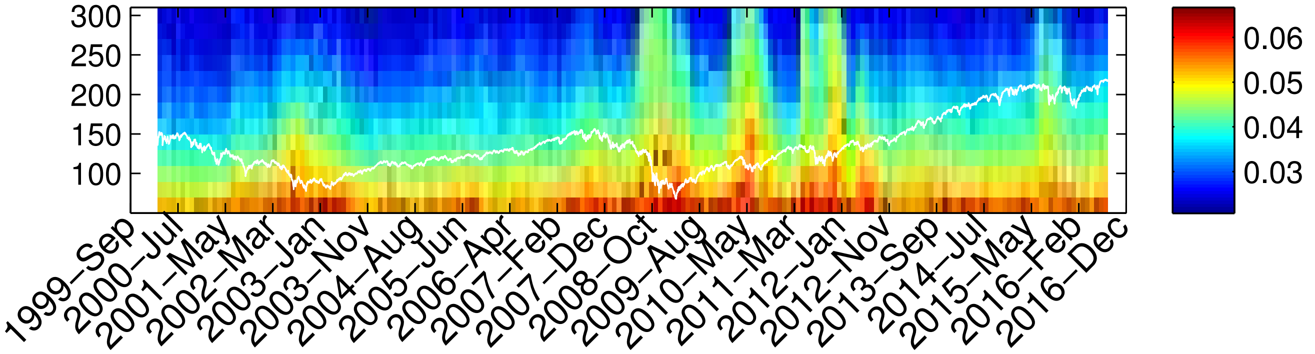

c)

Figure 8. Critical point (maximum points of susceptibility curves) as a) mean degree and b) threshold level. c) deviation of susceptibility curves from its maximum. Horizontal dimension corresponds to different time periods (with time window = 120 working days) and vertical dimension corresponds to different sub-sample sizes. S&P500 index is scaled and plotted (with white color) as a indicator of financial crises.

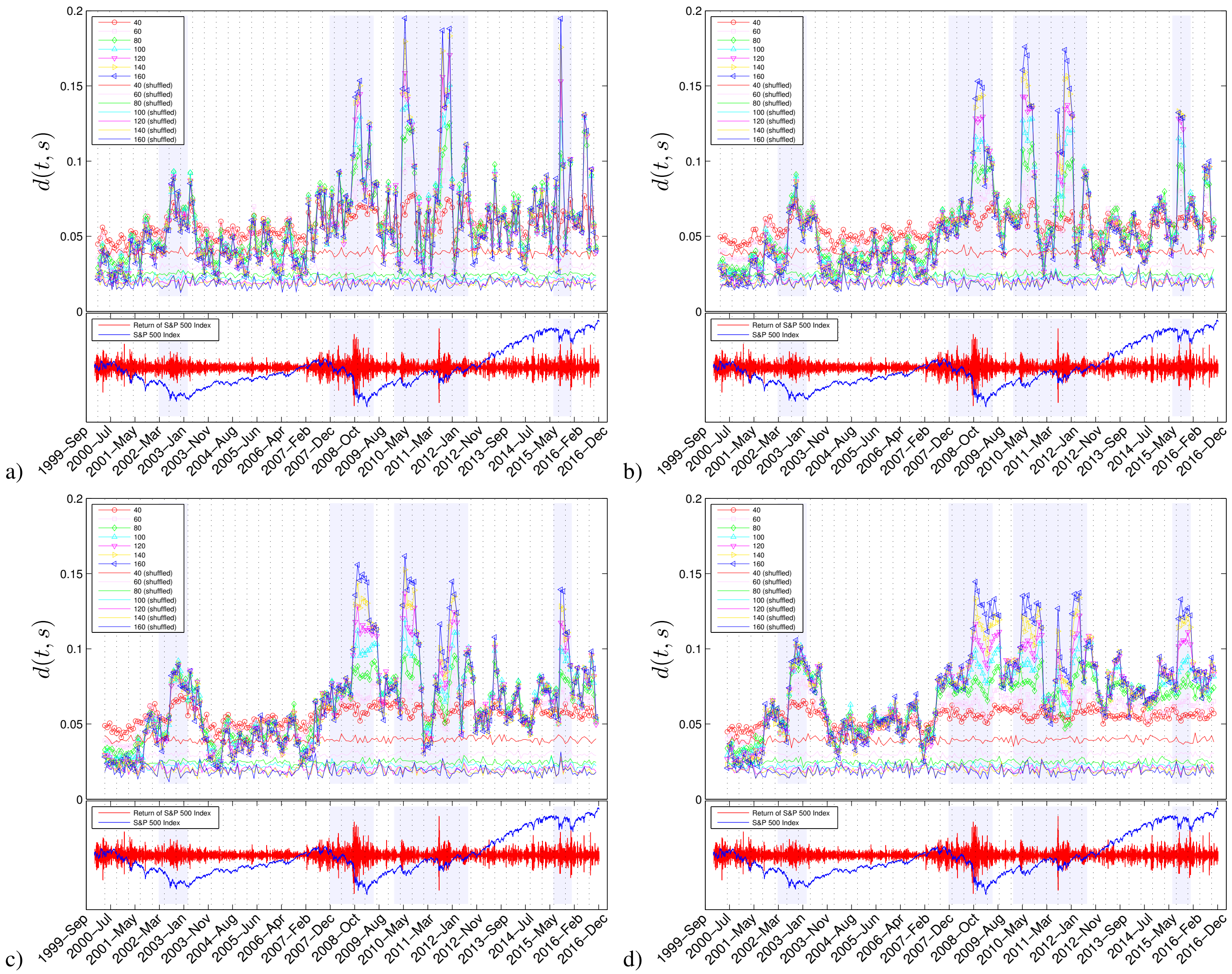

The mean absolute difference between theory and our computations for the giant component probabilities (top panel in each figure) for different time periods a) $\tau = 30$, b) $\tau = 60$, c) $\tau = 90$ and d) $\tau = 120$ and different sub-graph size (different symbols of different colors). Larger sub-graph sizes exhibit larger fluctuations in time. The thin solid curves in different colors show the same quantity for the corresponding shuffled network of each size which are comparatively less fluctuating around a mean value. The bottom panel in each figure shows the data for S&P500 index (blue curve) and its increments (red curve). The vertical bars in light blue show four different major crisis periods: (i) Stock market downturn of 2002, (ii) Financial crisis of 2007-08, (iii) 2010 Flash Crash and August 2011 stock markets fall, and (iv) 2015-16 stock market sell off.
